# Piezo1 promoted hepatocellular carcinoma progression and EMT through activating TGF-β signaling by recruiting Rab5c

**DOI:** 10.1186/s12935-022-02574-2

**Published:** 2022-04-23

**Authors:** Yi-ming Li, Cong Xu, Bo Sun, Fang-jing Zhong, Momo Cao, Lian-yue Yang

**Affiliations:** Liver Cancer Laboratory, Department of Surgery, Xiangya Hospital, Central South University, Xiangya Road 87, Changsha, 410008 Hunan China

**Keywords:** Piezo1, Hepatocellular carcinoma, Prognosis, Cancer progression, TGF-β signaling, EMT

## Abstract

**Background:**

Piezo1 has been revealed to play a regulatory role in vascular development and progression of variety tumors. However, whether and how the progression of hepatocellular carcinoma (HCC) regulated by Piezo1 remains elusive. This study aimed to elucidate the effect and mechanisms of Piezo1 in HCC.

**Methods:**

The mRNA and protein expression level of Piezo1 in HCC samples and cell lines was determined by qRT-PCR, western blot and immunohistochemistry analyses. Two independent study cohorts containing 280 patients were analyzed to reveal the association between Piezo1 expression and clinicopathological characteristics. Series of in vitro and in vivo experiments were used to validate the function of Piezo1 in HCC. Gene set enrichment analysis (GSEA) was performed to explore the signaling pathway of Piezo1. Immunoprecipitation, immunofluorescence and in vitro and in vivo experiments were used to explore the molecular mechanism of Piezo1 in HCC progression.

**Results:**

Our results demonstrated the Piezo1 expression was significantly upregulated in HCC tissues and cell lines, and upregulation of Piezo1 closely correlated with aggressive clinicopathological features and poor prognosis. Knockdown of Piezo1 in HCCLM3 and Hep3B cells significantly restrained proliferation, migration, invasion and epithelial–mesenchymal transition (EMT) of HCC cells in vitro, and tumor growth, metastasis, EMT in vivo. TGF-β signaling pathway was most significant enriched pathway in GSEA. Finally, tumor promotion effect of Piezo1 was found to exerted through recruiting and combining Rab5c to activating TGF-β signaling pathway.

**Conclusions:**

Piezo1 significantly related to poor prognosis and promotes progression of hepatocellular carcinoma via activating TGF-β signaling, which suggesting that Piezo1 may serve as a novel prognostic predictor and the potential therapeutic target for HCC patients.

**Supplementary Information:**

The online version contains supplementary material available at 10.1186/s12935-022-02574-2.

## Introduction

Hepatocellular carcinoma (HCC) ranks as the sixth most common malignant tumor and the fourth leading cause of cancer death worldwide, and China is one the most high risk HCC area [1]. Even multiple therapeutic measures have been proposed in newly presented guideline of HCC in China [2], such as local ablation, surgical resection or liver transplantation can be curative treatment options for early-stage HCC, liver resection remains the most effective approach for early HCC [3]. Survival benefits have obtained from chemoembolization for intermediate HCC and sorafenib for advanced HCC [4]. Although its mortality decreased along with advances in surgical resection or liver transplantation, the long-term outcome and effect of medicine remain unsatisfactory [5]. The 5-year survival rate in HCC patients beyond the Milan criteria after surgical resection is only 30% to 50% [6, 7], and median survival ranging from 2.5 to 10.6 months without effective treatment [8–10]. As the standard treatment for patients with advanced HCC, Sorafenib could just extended the median overall survival to 12.3 moth, mainly due to the high frequency of recurrence and metastasis. Comprehensive understanding of the molecular mechanisms underlying the Progression of HCC is crucial for its prevention, diagnosis and treatment [11]. Up to now, many factors have been identified to play important role in the progress of HCC, including aberrantly expressed miRNAs, LncRNAs and proteins, and has been suggested to be the HCC prognostic or diagnostic markers [12–15]. Although remarkable improvements had been done by global scientists. However, the molecular mechanisms of HCC progression remain largely unclear.

The Piezo family are known as mechanosensitive cation selective channels includes two isoforms, Piezo1 and Piezo2 [16]. Piezo1 is widely expressed in numerous mammalian tissues with particularly high in lung, bladder and skin [17–19]. In addition to be a mechano-sensors and converts environmental signals into intracellular Ca^2+^ responses, and involved in vascular development and function [20]. Recent studies have addressed that Piezo1 also have multiple functions, in regulating the cardiac macrovascular development during early embryogenesis, control rapid epithelial cell division, lymphatic valve formation and altered neuron stem cells differentiation [21–24]. So far, Piezo1 were involved in proliferation and migration in gastric cancer, breast cancer, synovial sarcoma and glioma [25–28], but the function and mechanism of Piezo1 in HCC remain poorly elucidated. In our preliminary study, Piezo1 highly expressed in HCC tissue and cell lines, but not Piezo2. Thus, those studies led to a hypothesis that Piezo1 plays an important role in HCC progression.

In this study, the function of Piezo1 was explored in HCC and found that high expression of Piezo1 is closely correlated with poor prognosis of HCC patients. We also confirmed that Piezo1 promotes HCC progression through EMT. Mechanism studies show that Piezo1 could recruits and activates Rab5c in HCC, which promoted the phosphorylation of Smad2/3 and triggers classical TGF-β signaling pathway in HCC. Thus, Piezo1 might serve as a potential prognostic biomarker and therapeutic target for HCC.

## Materials and methods

### HCC samples and patients

A total of 150 HCC specimens in training cohort collected from January 2009 to December 2012 were randomly selected from the patients received liver resection at Department of Surgery, Xiangya Hospital of Central South University. Another 130 HCC specimens in validation cohort collected from January 2010 to December 2012 were randomly selected from the Department of Abdominal Surgical Oncology, Affiliated Cancer Hospital of Xiangya School of Medicine, Central South University. The patient demographics and clinicopathological variables of the two cohorts are described in Additional file 3: Table S1. Furthermore, 30 matched fresh HCC tissues and adjacent nontumoral liver tissues (ANLTs) were collected from Xiangya Hospital from September to December 2019. 10 fresh HCC tissues in each clinic subtypes were collected from Xiangya Hospital from June to December 2019. The diagnosis of HCC in all patients was confirmed by two independent histopathologists. The present study was approved by the Ethics Committee of Xiangya Hospital, Central South University. All patients and their families provided written informed consent and agreed to the use of their tissue samples in the study in accordance with the Declaration of Helsinki.

### Extraction of membrane and cytosol fraction

Cells were washed 3 times by PBS and added lysis buffer (250 mM sucrose, 10 mM HEPES, 1 mM EDTA, pH 7.5) supplemented with protease inhibitors, and scraped into centrifuge tube. Extracts were mechanically lysed 50times by homogeniser, and subjected to 90 min of ultracentrifugation at 45,000 rpm (Thermo Fisher Scientific, Waltham, MA) at 4 °C. The resulting supernatant was the cytosolic fraction. The membrane fraction was resuspended in 100 μl immunoprecipitation assay buffer (10 mM Tris–HCl, pH 7.5, 1 mM EDTA, 0.5 mM EGTA, 1% Triton X-100, 0.1% sodium deoxycholate, 0.1% SDS 140 mM NaCl), with protease inhibitors. Cytosol and membrane protein bands were quantified and relativized against their respective fraction markers RhoGDI and transferrin receptor [29].

### Immunohistochemical (IHC) analysis and scoring

Immunohistochemical staining on formalin-fixed, paraffin-embedded tissue sections 4 μminthickness wasperformed using the polymer HRP detection system (Zhongshan Goldenbridge Biotechnology, Beijing, China). Immunohistochemical experiments were conducted as previously described [14, 30]. The IHC score of target proteins was independently evaluated by two investigators according to the proportion and intensity of positive cells within five randomly selected fields per slide (magnification, × 400). The intensity was assessed by four grades: 0 for none; 1 for weak; 2 for moderate; 3 for strong. The percentage of positive cells was divided into five degrees: 0, no positive tumor cells; 1 for ≤ 5%; 2 for 6–25%; 3 for 26–75%; 4 for ≥ 76%. Immunoreactive score was calculated by multiplying the staining extent score with the intensity score. As previous reported, high expression was defined as a staining index score > 4, while low expression was defined as a staining index score ≤ 4[31, 32].

### HCC mouse models

Animal xenograft assays were conducted with 6-weekold male BALB/c nude mice (six mice per group). 5 × 10^6^ indicated cells were subcutaneously injected into the right dorsal flank of nude mice. Tumor sizes were measured at the indicated time points and calculated with the following formula: Tumor volume = L × W^2^ × 0.5 (L, length; W, width) [12]. After 4 weeks, the mice were sacrificed, and the tumors were harvested to weigh and undergo further experiments. Orthotopic tumor implantation was performed as described previously [33]. After 8 weeks, the mice were sacrificed, and the livers and lungs were harvested, the tumor size was measured by the vernier caliper as previously described [12], imaged and processed for histopathological examination. All animal experiments were conducted at the Animal Institute of CSU according to the protocols approved by the Medical Experimental Animal Care Commission of CSU. As the proposal of the Animal Institute of CSU, the euthanasia of all the experimental mice were used the pentobarbital sodium, 150 mg/Kg, intraperitoneal injection.

### Statistical analysis

Statistical analysis was performed using SPSS 24.0 software (SPSS Inc., Chicago, IL). The experimental data was presented as the mean ± SD and analyzed using Student’s* t* test or one-way ANOVA. The Chi-squared test was applied to examine the association between Piezo1 expression and clinicopathological parameters. Survival curves for patients were calculated using the Kaplan–Meier method and analyzed using the log-rank test. Prognostic factors were examined by univariate and multivariate analyses using the Cox proportional hazards model. Spearman’s rank analysis was performed to determine the correlation between different protein levels. Student’s* t* test was performed to measure the membrane and cytolic protein expression level. All differences were deemed statistically significant at *P* < 0.05.

Further details of materials and methods are described in the Additional file 1: Materials and methods.

## Result

### Piezo1 is significantly upregulated in HCC cell lines and tissues

**Fig. 1:**
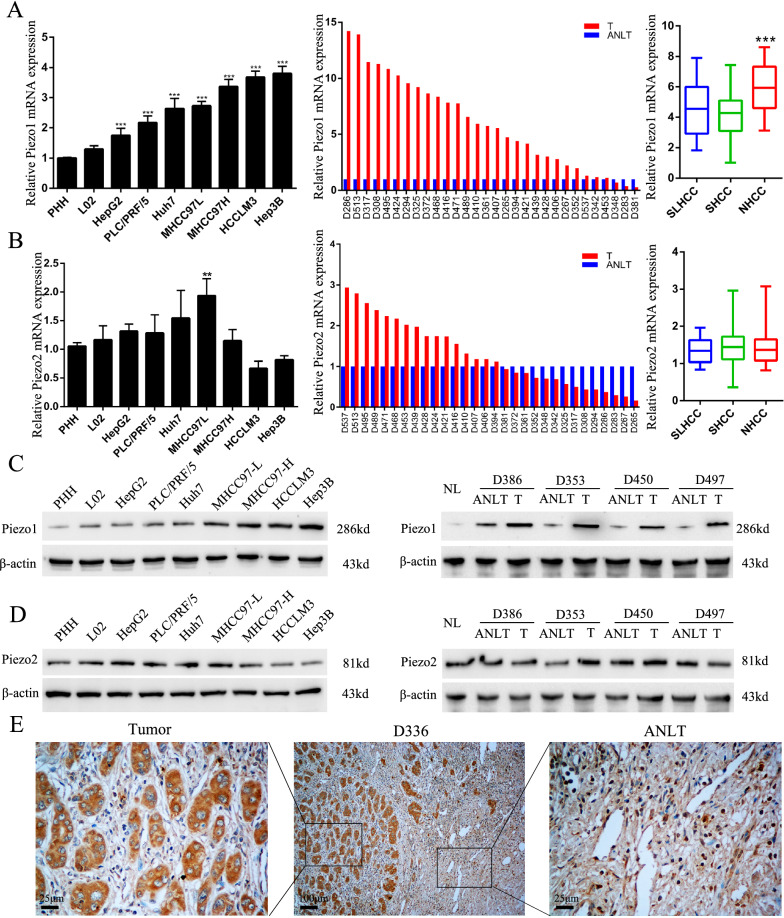
Piezo1 is significantly upregulated in HCC cell lines and tissues. **A** qRT-PCR identified the mRNA of Piezo1 in seven HCC cell lines, PHH and L02 cells (n = 3), 30 pairs of fresh HCC tissues and ANLTs, and 3 clinical subtypes of HCC tissues (15 cases each group). Data are shown as mean ± SD and analyzed using Student’s t test. **B** qRT-PCR identified the mRNA of Piezo2 in HCC cell lines and tissues. **C** Piezo1 protein expression levels were detected in seven HCC cell lines, PHH and L02 cells, in normal liver tissue and paired fresh HCC tissues and ANLTs by Western blot. **D** Piezo2 protein expression levels were detected in in HCC cell lines and tissues. **E** Representative IHC images of Piezo1 expression in HCC tumor tissue and ANLTs. *PHH* primary human hepatocytes, *SLHCC* solitary large HCC, *SHCC* small HCC, *NHCC* nodular HCC, *NL* normal liver, *T* HCC tissue, *ANLT* adjusted non-tumor liver tissue. *P < 0.05; **P < 0.01; ***P < 0.001

Firstly, we examined the levels of Piezo1 and Piezo2 mRNA in 7 HCC cell lines and 2 normal hepatocytes, the Primary human hepatocytes (PHH) and immortalized hepatocytes (L02) (Fig. 1A). Notably, the expression of Piezo1 mRNA in HCC cells significantly stronger than PHH and L02. But this difference of expression was not observed in Piezo2 (Fig. 1B). Then, we also detected the mRNA expression of Piezo1 and Piezo2 in frozen HCC tissue and the corresponding adjusted nontumor liver tissue (ANLT). Consistently, mRNA expression level of Piezo1 significantly upregulated compared with ANLT, but the expression difference was not detected in Piezo2. Previously, we found a new clinic subtype of HCC. We summarized this subtype as Solitary Large HCC (SLHCC) which tumor major axis > 5 cm, expansive growth, and with intact capsule or pseudocapsule [6]. According to these clinic pathologic features, we classified HCC into 3 clinic subtypes, the SLHCC, nodular HCC (NHCC, node number > 1) and Small HCC (SHCC, tumor diameter ≤ 5 cm). In our previous research, the exhibited a similar long-term overall and disease-free survival with SHCC, but much better than NHCC [4, 7–9]. We also detected mRNA of Piezo1 in the 3 clinic subtypes of HCC, and the NHCC with high metastatic potentials expressed relatively higher level of Piezo1 than the SLHCC and SHCC with low metastatic potentials, but Piezo2 was not significantly differential expressed. Then, Piezo1 and Piezo2 protein expression in HCC cell lines and PHH, L02 cells was detected by WB (Fig. 1C, D, the quantification result seen in Additional file 2: Fig. S1). Therefore, we supposed that Piezo1 might associate with progression of HCC, but not Piezo2. Expression level of Piezo1 was also analyzed by immunohistochemistry (IHC), which suggested that Piezo1 protein was highly expressed in HCC (Fig. 1E). These data reveal that Piezo1 upregulated in HCC cell lines and tissues, and indicates that it’s very necessary to explore the role of Piezo1 in HCC progression.

### Upregulated Piezo1 is associated with HCC poor prognosis

**Fig. 2:**
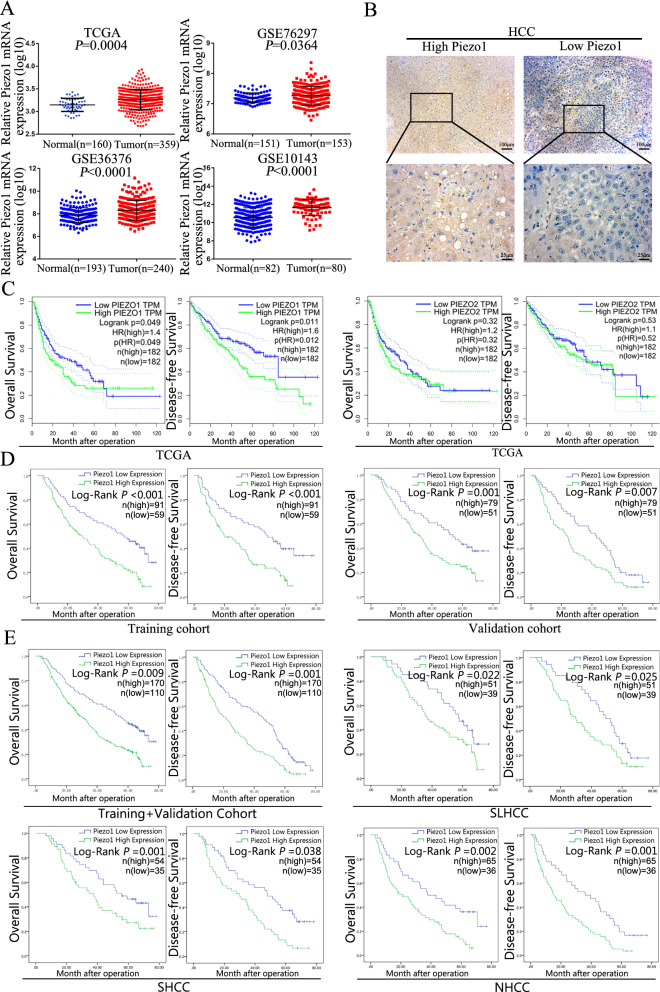
Upregulated Piezo1 is associated with HCC poor prognosis. **A** Piezo1 expression was higher in HCC tissues than Normal liver tissues according to the analysis of data from TCGA and GEO (GSE76297, GSE36376, GSE10143) using Student’s t test. **B** Representative images of low Piezo1 expression cases and high Piezo1 expression cases were shown. Magnification, × 100, × 400. **C** Kaplan–Meier analysis of OS and DFS based on Piezo1 and Piezo2 mRNA expression in data from TCGA. **D** Kaplan–Meier analysis of OS and DFS with high or low Piezo1 expression in Training and Validation Cohort. **E** The OS and DFS of HCC patients in the cohort integrated by Training and Validation cohort. The survival curve was calculated with the log-rank test in SLHCC, SHCC, and NHCC, the three clinical HCC subtypes

After the upregulated expression level of Piezo1 in HCC was determined, we further verified the expression difference through the analyses of mRNA datasets from TCGA (*P* = 0.0004) and Gene Expression Omnibus (Fig. 2A, GSE76297 *P* = 0.0364, GSE36376 *P* < 0.0001, GSE10143 *P* < 0.0001), which verified our experiments that Piezo1 upregulated in HCC. In order to explore the correlation between Piezo1 expression level and clinic pathologic features of HCC, we performed IHC in 280 patients collected form 2 hospitals, and concluded to Training cohort and Validation cohort (Fig. 2B). Before the research, the comparability of data was verified (Additional file 3: Table S1). Then the correlation test was performed and revealed the Piezo1 expression level correlated with several fatal clinicopathological features. In the training cohort (n = 150), a high expression level of Piezo1 was closely correlated with tumor size, tumor nodular number, capsulation formation, high Edmondson–Steiner grade, micro- and macro-vascular invasion, advanced tumor node metastasis stage (TNM), Barcelona Clinic Liver Cancer (BCLC) stage and China Clinic Liver Cancer (CNLC) stage [2] (all *P* < 0.05, Table 1), and verified by Validation cohort. Furthermore, uni- and multi-variate analysis revealed that high Piezo1 expression was an independent risk factor for both OS and DFS of HCC patients after liver resection (Table 2), this result was verified in validation cohort (Additional file 3: Table S2). In addition, Kaplan–Meier survival analysis in TCGA showed that HCC patients with low Piezo1 had longer OS and DFS, but Piezo2 showed no significance between low- and high- expression group (Fig. 2C), which consistent with our above research. Notably, in training cohort, survival analysis revealed that the high Piezo1 expression group had worse OS (1-, 3-, and 5-year OS: 91.33%, 67.33%, and 48.67% *vs*. 79.33%, 41.33%, and 21.33%) and DFS rates (1-, 3-, and 5-year DFS: 82.31%, 61.54%, and 41.54% vs. 70.77%, 31.54%, and 10.77%) than patients in the low-expression group (Fig. 2D), and also verified in Validation cohort. Besides, this result was also observed in integrated cohort combined by training and validation cohort and 3 clinical subtypes of HCC (Fig. 2E). These data fully confirmed that Piezo1 expression level was closely correlated with clinic pathologic features and poor survival, and has the potential to be a novel independent prognosis biomarker for HCC patients after hepatic resection.

**Table 1:** Correlation between Piezo1 expression and clinicopathologic characteristics of HCC patients in training cohort and validation cohort

Clinicopathologic variables	Training cohort				Validation cohort			
	n	Piezo1 expression		*P*	n	Piezo1 expression		*P*
		High	Low			High	Low	
Gender				0.190				0.438
Female	42	29	13		31	17	14	
Male	108	62	46		99	62	37	
Age (years)				0.220				0.238
≤ 50	67	37	30		63	35	28	
> 50	83	54	29		67	44	23	
AFP(ng/ml)				0.266				0.431
< 20	36	19	17		38	22	16	
≥ 20	114	72	42		92	57	35	
Hepatitis B status				0.688				0.496
Negative	43	25	18		48	31	17	
Positive	107	66	41		82	48	34	
Liver cirrhosis				0.196				0.605
Absent	69	38	31		55	32	23	
Present	81	53	28		75	47	28	
Child–Pugh classification				0.587				0.416
A	98	61	37		81	42	39	
B	52	30	22		49	29	20	
Tumor size(cm)				**0.024**				**0.020**
≤ 5	57	28	29		55	27	28	
> 5	93	63	30		75	52	23	
Tumor nodule number				**0.004**				**0.027**
Solitary	72	35	37		71	37	34	
Multiple (≥ 2)	78	56	22		59	42	17	
Capsulation formation				**0.014**				**0.004**
Presence	58	28	30		56	26	30	
Absence	92	63	29		74	53	21	
Edmondson-Steiner grade				**0.004**				**0.013**
I&II	62	29	33		54	26	28	
III&IV	88	62	26		76	53	23	
Microvascular invasion				**0.034**				**0.016**
Absence	91	49	42		83	44	39	
Presence	59	42	17		47	35	12	
Macrovascular invasion				**0.032**				**0.027**
Absence	122	69	53		108	61	47	
Presence	28	22	6		22	18	4	
BCLC stage				**0.003**				**0.044**
0&A	43	18	25		45	22	23	
B&C	107	73	34		85	57	28	
TNM stage				**0.004**				**0.029**
I	62	29	33		56	28	28	
II&III	88	62	26		74	51	23	
CNLC stage				**0.037**				**0.003**
I	86	46	40		81	41	40	
II&III	64	45	19		49	38	11	

Bold values *P* < 0.05

*AFP* alpha-fetoprotein, *TNM* tumor node metastasis, *BCLC* Barcelona Clinic Liver Cancer, *CNLC* China Clinic Liver Cancer

**Table 2:** Univariate and multivariate analyses of risk factors associated with overall survival and disease-free survival of HCC patients in training cohort

Clinicopathologic variables	OS		DFS	
	Univariate analysis	Multivariate analysis	Univariate analysis	Multivariate analysis
	*P* HR (95% CI)	*P* HR (95% CI)	*P* HR (95% CI)	*P* HR (95% CI)
Gender	0.221	NA	0.245	NA
Female	1		1	
Male	1.295(0.856–1.958)		1.278(0.845–1.931)	
Age (years)	**0.018**	0.325	**0.014**	0.113
≤ 50	**1**	1	**1**	1
> 50	**1.595(1.085–2.345)**	1.237(0.810–1.889)	**1.622(1.102–2.385)**	1.366(0.929–2.008)
AFP(ng/ml)	**0.001**	**0.003**	**0.002**	** < 0.001**
< 20	**1**	**1**	**1**	**1**
≥ 20	**2.235(1.384–3.611)**	**2.198(1.300–3.713)**	**2.154(1.334–3.478)**	**2.308(1.441–3.696)**
Hepatitis B status	0.655	NA	0.634	NA
Negative	1		1	
Positive	1.099(0.727–1.660)		1.106(0.732–1.671)	
Liver cirrhosis	0.266	NA	0.350	NA
Absent	1		1	
Present	1.239(0.849–1.809)		1.196(0.822–1.739)	
Child–Pugh classification	0.480	NA	0.468	NA
A	1		1	
B	1.148(0.783–1.684)		1.153(0.786–1.691)	
Tumor size (cm)	0.245	NA	0.267	NA
≤ 5	1		1	
> 5	1.248(0.859–1.814)		1.235(0.850–1.795)	
Tumor nodule number	**0.001**	**0.001**	**0.001**	**0.002**
Solitary	**1**	**1**	**1**	**1**
Multiple (≥ 2)	**1.935(1.329–2.817)**	**2.195(1.379–3.493)**	**1.864(1.282–2.710)**	**1.983(1.296–3.034)**
Capsulation formation	**0.008**	0.263	**0.010**	0.214
Presence	**1**	1	**1**	1
Absence	**1.650(1.139–2.390)**	1.288(0.827–2.005)	**1.619(1.120–2.342)**	1.298(0.860–1.960)
Edmondson- Steiner grade	**0.004**	0.504	**0.001**	0.193
I&II	**1**	1	**1**	1
III&IV	**1.784(1.207–2.638)**	1.253(0.647–2.427)	**1.790(1.253–2.557)**	1.308(0.873–1.958)
Microvascular invasion	**0.027**	**0.041**	** < 0.001**	**0.010**
Absence	**1**	**1**	**1**	**1**
Presence	**1.529(1.049–2.229)**	**1.898(1.028–3.507)**	**2.110(1.489–2.991)**	**2.044(1.187–3.522)**
Macrovascular invasion	** < 0.001**	**0.008**	** < 0.001**	**0.012**
Absence	**1**	**1**	**1**	**1**
Presence	**2.728(1.741–4.273)**	**2.191(1.230–3.902)**	**2.247(1.450–3.483)**	**2.086(1.174–3.707)**
BCLC stage	**0.001**	**0.012**	**0.001**	**0.001**
0&A	**1**	**1**	**1**	**1**
B&C	**1.914(1.301–2.815)**	**1.697(1.125–2.560)**	**1.828(1.289–2.592)**	**1.873(1.286–2.729)**
TNM stage	** < 0.001**	**0.010**	** < 0.001**	**0.005**
I	**1**	**1**	**1**	**1**
II&III	**2.197(1.481–3.259)**	**1.778(1.148–2.755)**	**1.975(1.387–2.813)**	**1.797(1.196–2.700)**
CNLC stage	** < 0.001**	**0.001**	**0.008**	**0.045**
I	**1**	**1**	**1**	**1**
II&III	**2.300(1.576–3.358)**	**2.019(1.318–3.094)**	**1.593(1.130–2.246)**	**2.002(1.017–3.942)**
Piezo1 expression	** < 0.001**	**0.026**	** < 0.001**	**0.001**
Low	**1**	**1**	**1**	**1**
High	**2.048(1.374–3.053)**	**1.671(1.064–2.625)**	**1.943(1.355–2.787)**	**1.894(1.285–2.972)**

Bold values *P* < 0.05

*HR* hazard risk ratio, *CI* confidence interval, *NA* not applicable, *AFP* alpha-fetoprotein, *HBsAg* hepatitis B surface antigen, *TNM* tumor node metastasis, *BCLC* Barcelona Clinic Liver Cancer, *CNLC* China Clinic Liver Cancer

### Piezo1 promotes HCC proliferation, invasion and metastasis in vitro and in vivo

**Fig. 3:**
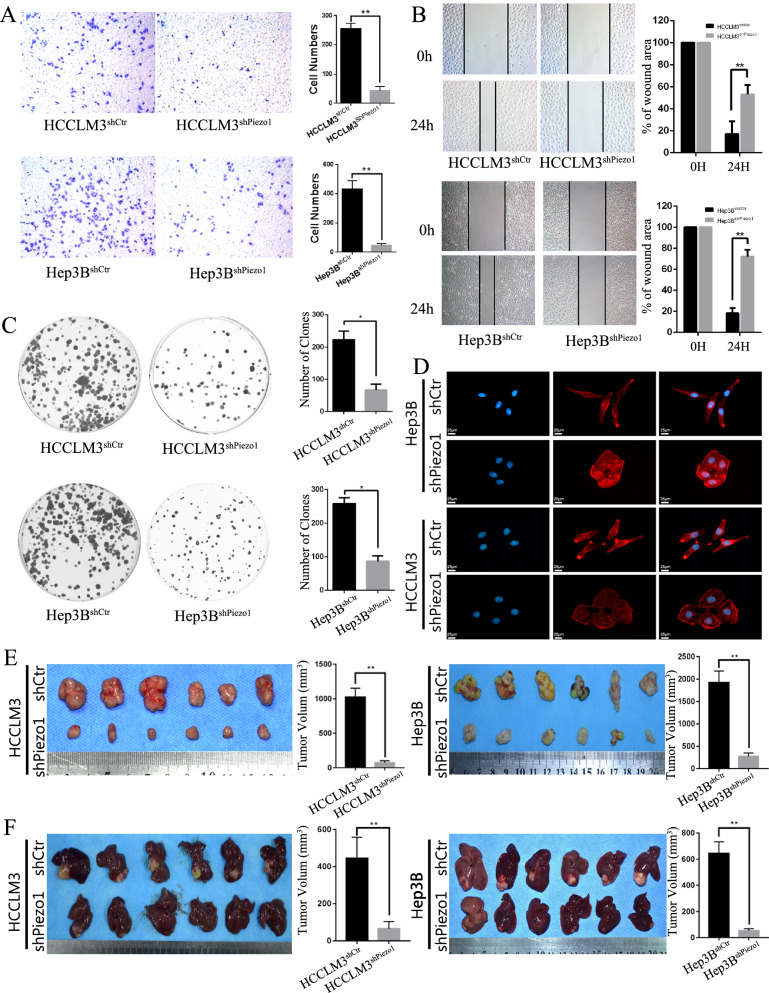
Piezo1 promotes HCC invasion, proliferation and metastasis in vitro and in vivo. **A** Transwell invasion assay was used to detect the invasive capacities of HCCLM3^shPiezo1^, Hep3B^shPiezo1^ and the corresponding control cells (n = 6 for each group). **B** Wound healing assay was used to detect the migratory capacities of Piezo1 interfered HCCLM3 and Hep3B cells(n = 6). **C** Colony formation assays was used to detect the proliferation of Hep3B^shPiezo1^, HCCLM3^shPiezo1^ and the control HCC cells (n = 6 for each group). **D** Representative images of the cytoskeleton showed that Piezo1 affected the cellular morphology. **E** Subcutaneous tumors from HCCLM3^shPiezo1^ and Hep3B^shPiezo1^ cells and their control cells. Tumor volumes are shown in the right panels (n = 6 for each group). **F** Orthotopic tumors from HCCLM3^shPiezo1^ and Hep3B^shPiezo1^ cells and their control cells. Tumor weight are shown in the right panels (n = 6 for each group). *P < 0.05; **P < 0.01, the difference between groups analyzed using Student’s t test

To understand the function of Piezo1 in HCC cells, we manipulated Piezo1 expression in cells by short hairpin RNA (shRNA). Due to the molecular weight of Piezo1 is too large to construct overexpression vector, we interfered Piezo1 in two highest expression cell lines HCCLM3 and Hep3B cells by three shRNAs separately, as named HCCLM3^shPiezo1^ and Hep3B^shPiezo1^. The corresponding control lentivirus transfected HCCLM3 and Hep3B, as named HCCLM3^shCtr^ and Hep3B^shCtr^. The expression level of Piezo1 was identified by real-time PCR and western blotting. shRNA2 was the most effective one and was chosen for further study (Additional file 2: Fig. S2A, B). The wound-healing and transwell assays were used to investigate migration and invasion capacity. The results showed that HCCLM3^shCtr^ cells had a faster wound closure rate and more invasion than HCCLM3^shPiezo1^ cells, and Hep3B^shPiezo1^ cells had also markedly reduced migratory and invasive capacity (Fig. 3A, B). Compared to HCCLM3^shPiezo1^, cell clone formation assay (Fig. 3C) and MTT assay (Additional file 2: Fig. S3) indicated HCCLM3^shCtr^ had a higher proliferation rate. Consistently, Hep3B^shCtr^ cells also formed more colonies in colony formation assay. In IF stained F-actin (Fig. 3D), we also noticed the morphological change when Piezo1 was interfered, in which the spindle-like mesenchymal morphology of HCCLM3^shCtr^ and Hep3B^shCtr^ changed into cobblestone-like epithelial morphology of HCCLM3^shPiezo1^ cells and Hep3B^shPiezo1^ cells. To verify the above findings in vivo, we established subcutaneous (SC) xenograft tumor and orthotopic xenograft tumor models, as previously described [34]. After 6 weeks, HCCLM3^shCtr^ and Hep3B^shCtr^ cell-derived tumors at the SC implantation sites were larger and grew more rapidly than HCCLM3^shPiezo1^ and Hep3B^shPiezo1^ derived tumors (Fig. 3E, and the growth curve was shown in Additional file 2: Fig. S4 and tumor weight in Additional file 2: Fig. S5). Consistently, liver orthotopic xenograft tumor also showed that HCCLM3^shPiezo1^ and Hep3B^shPiezo1^ derived tumors significantly smaller than HCCLM3^NC^ and Hep3B^NC^-derived tumors. Ki67 for the subcutaneous xenograft tumors and orthotopic xenograft tumors was detected by IHC (Additional file 2: Fig. S6), which indicates that Ki67 negative in Piezo1 interfered HCCLM3 and Hep3B cells and positive in the control cells. All these results indicate that Piezo1 knockdown inhibited tumor growth in vivo (Fig. 3F). Taking these results together, our results shows that Piezo1 promotes HCC growth, progression in vitro and in vivo.

### Piezo1 promotes EMT in HCC

**Fig. 4:**
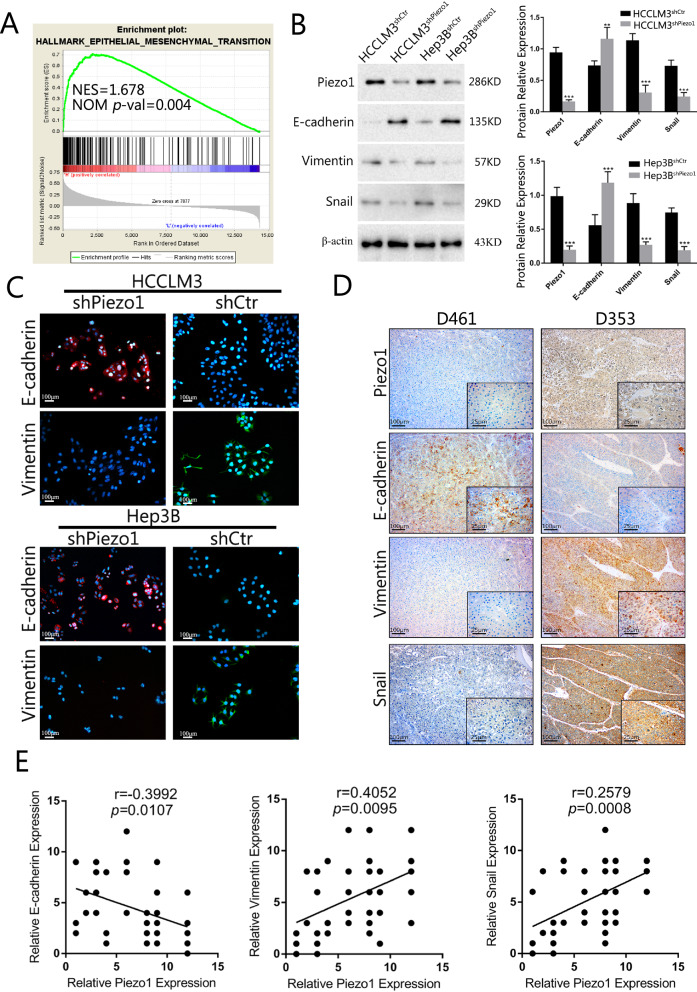
Piezo1 promotes EMT in HCC. **A** GSEA of Piezo1 expression in HCC patients from the TCGA database revealed that the EMT signature is significantly positively associated with Piezo1 expression (NES = 1.678, NOM *p*-val = 0.004). **B** Piezo1-mediated expression levels of EMT markers were detected by western blot. The right panel showed the result of semi-quantitative analysis of western bolt, analyzed using Student’s t test. **C** Double immunofluorescence staining showed that Piezo1 affected cellular expression of EMT markers (Red: E-cadherin; Green: Vimentin). **D** Representative IHC images of Piezo1, E-cadherin, vimentin and Snail expression in HCC tissue. **E** Piezo1 and EMT markers expression correlations were analyzed by Spearman rank correlation tests in the Clinical samples (n = 40). **P < 0.01; ***P < 0.001

In the present study, the morphological changes in IF stained F-actin attracted our attention when we silenced Piezo1 expression in HCCLM3 and Hep3B cells. In addition, the hallmarks of EMT enriched in high Piezo1 group in the gene set enrichment analysis (GSEA) of TCGA database (NES = 1.678, NOM *p*-val = 0.004, Fig. 4A). We speculated that Piezo1 promoted HCC progression through EMT. To confirm our hypothesis, cytological and histological experiments were conducted to verify the expression relationship between Piezo1 and EMT markers. At the protein level, Piezo1 knockdown in HCCLM3 and Hep3B cells resulted in the expression of E-cadherin increased and vimentin decreased, whereas high Piezo1 expressed HCCLM3^shCtr^ and Hep3B^shCtr^ cells resulted in opposing results (Fig. 4B). IF analysis showed that the expression of the mesenchymal marker vimentin was significantly reduced in Piezo1 interfered HCCLM3^shPiezo1^ and Hep3B^shPiezo1^ cells and increased the expression of the epithelial marker E-cadherin, while relatively high expression level of Piezo1 induced inverse results in HCCLM3^shCtr^ and Hep3B^shCtr^ (Fig. 4C). IHC staining of consecutive HCC sections also showed that vimentin expression levels were up-regulated in high Piezo1-expressing cell-derived tumors, whereas E-cadherin expression was reduced (Fig. 4D). Moreover, IHC revealed that Piezo1 expression was negatively correlated with E-cadherin expression and positively correlated with vimentin expression (Fig. 4E). These results indicated that Piezo1 promotes EMT in HCC cell lines.

### Piezo1 promotes HCC progression via TGF-β signaling

**Fig. 5:**
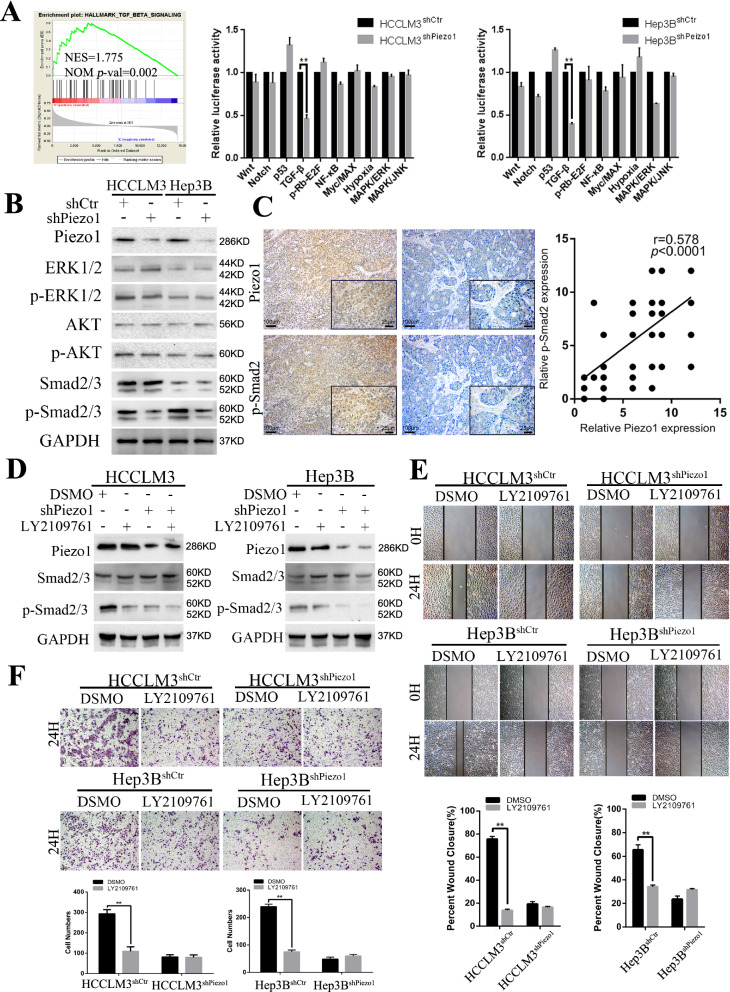
Piezo1 promotes HCC growth and metastasis via TGF-β signaling. **A** GSEA of Piezo1 expression in HCC patients from the TCGA database revealed that the TGF-β signaling is significantly positively associated with Piezo1 expression (NES = 1.775, NOM *p*-val = 0.002). The right panels of 10-Pathway Reporter Array showed the TGF-β signaling significantly changed in Piezo1 interfered cells (n = 3), analyzed using Student’s t test. **B** The protein expression levels of Piezo1 and markers of TGF-β signaling were detected by Western blot. **C** Representative IHC images of Piezo1 and p-Smad2, their expression correlations were analyzed by Spearman rank correlation tests in 40 HCC tissue. **D** Smad2/3 and p-Smad2/3 in HCC cells after interfered Piezo1 or blocked TGF-β signaling (Blocked by LY2109761, the inhibitor of TGF-β pathway) were detected by WB. **E**–**F** Wound healing (**E**) and Transwell invasion (**F**) assays for HCCLM3^shPiezo1^, Hep3B^shPiezo1^ and their control cells with/without LY2109761 treatment (n = 6), **, *P* < 0.01, analyzed using Student’s t test

In order to investigate how the Piezo1 promotes EMT and progression of HCC, we turn to see potential signaling pathways manipulated by Piezo1 in HCC. Firstly, the GSEA of the TCGA database showed that TGF-β signaling was the most significantly enriched pathway in Piezo1 high group (NES = 1.775, NOM *p*-val = 0.002, Fig. 5A. The result of GSEA shown in Additional file 3: Tables S3, S4). Then, we conducted the Cignal Finder Cancer 10-Pathway Reporter Array (Qiagen, Germany) in Piezo1 expression interfered HCC cells to further screen and confirm the signaling pathway regulated by Piezo1. The results showed that TGF-β signaling was the most significantly altered pathway (Fig. 5A) between Piezo1 expression interfered HCC cells and the corresponding control cells. So, we further detected the expression of key members of the TGF-β signaling pathway in Piezo1 manipulated HCC cells, the result showed that in shPiezo1 cells, the protein levels of AKT, p-AKT, ERK, p-ERK and smad2/3 had no significant difference, only phosphorylated-smad2/3 was down-regulated in Piezo1 knockdown cells (Fig. 5B), which indicated the canonical TGF-β signaling. Meanwhile, several genes related to EMT were analyzed. The data showed that E-cadherin was up-regulated and vimentin was down-regulated in Piezo1 knockdown cells. Moreover, IHC staining in 40 HCC samples randomly selected from the training and validation cohorts showed that p-Smad2/3 protein was highly expressed in HCC tissues and positively correlated with Piezo1 expression (*P* < 0.001, r = 0.578, Fig. 5C). Then, the four cell lines were treated with LY2109761 (10 μM, 2 h), a TGF-β/smad2/3 inhibitor, and we found that LY2109761 did not affected Piezo1 expression. Compared with the control cells, HCCLM3^shPiezo1^ and Hep3B^shPiezo1^cells showed a decreasing trend in phosphorylation level of Smad2/3, which was similar to LY2109761 treated HCCLM3^shCtr^ and Hep3B^shCtr^ cells. It proves that Piezo1 activates TGF-β signaling in HCC cells (Fig. 5D). To further study the role of Piezo1 and TGF-β signaling in progression, Wound-healing (Fig. 5E) and transwell (Fig. 5F) assays showed that LY2109761 treatment obviously decreased the migration and invasion capacity of Piezo1 high expressed cells, but had none or less effect for cells in HCCLM3^shPiezo1^ and Hep3B^shPiezo1^cells which Piezo1 expression and TGF-β signaling activity was low. Taken together, the public database analysis and our results showed that Piezo1 could activate the canonical TGF-β/ Smad2/3 signaling induced EMT in HCC and, promoting HCC progression.

### Piezo1 activates TGF-β/Smad2/3 signaling by recruiting Rab5c

**Fig. 6:**
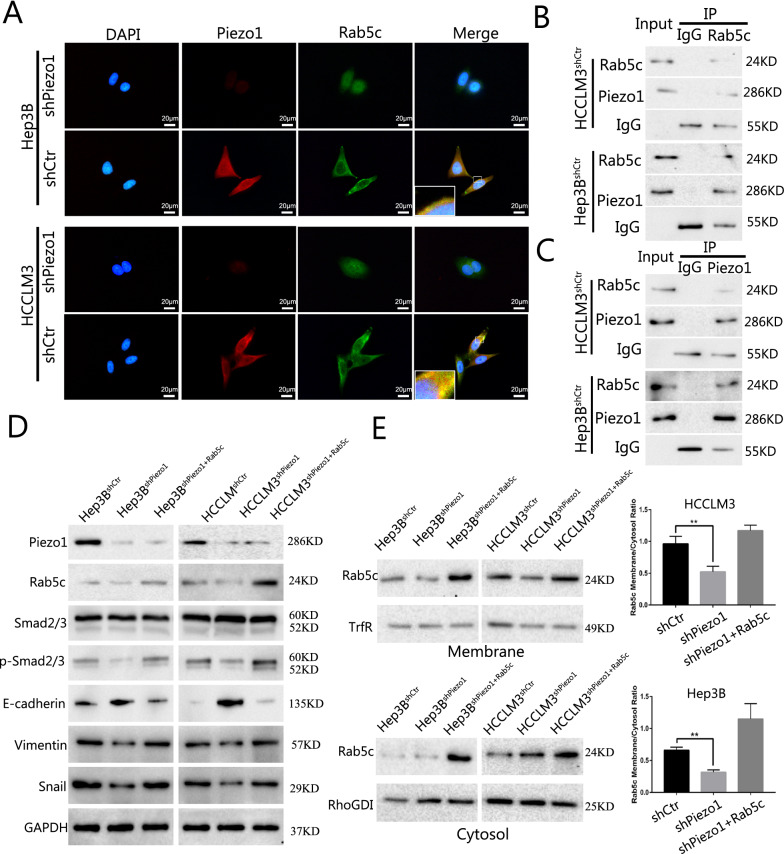
Piezo1 activates TGF-β/Smad2/3 signaling by recruiting Rab5c. **A** Rab5c tend to located at membrane with the presence of Piezo1 revealed by Immunofluorescence (IF) co-localization. **B** Piezo1 could interact with Rab5c in HCC cells analyzed by Co-immunoprecipitation assay. **C** Rab5c could combined with Piezo1 directly. **D** Protein expression level analyzed by western blot and showed that Piezo1 regulated the phosphorylation level of Smad2/3 and expression level of EMT markers, but not rab5c expression level. **E** Membrane enrichment analysis by Western blotting of RAB5c (N = 5, **p < 0.01). Membrane and cytosol fractions from HCC cells were obtained and analyzed by Western blot. Membrane and cytosol bands were relativized against their respective fraction marker, and membrane fractions were normalized against their corresponding cytosolic fraction and analyzed using Student’s t test

The above results revealed that Piezo1 promote HCC progression via activating the TGF-β signaling, to further explore the molecular mechanism by which Piezo1 exerted the functions in HCC. In consideration of that Piezo1 is a stretch-activated ion channel and the activation of Piezo1 channels increased the intracellular Ca^2+^ concentration, we treated HCCLM3 and Hep3B cells with Yoda1 (the activator of Piezo1, 20 mM) to increase the intracellular Ca^2+^ concentration and GsMTx4 (the inhibitor of Piezo1, 2.5 μM) to decrease Ca^2+^ concentration [19]^19^, then examined p-Smad2/3 and marker of EMT, found that Ca^2+^ influx was not the dominant factor in Piezo1 activated TGF-β signaling (Additional file 2: Fig. S7A, B). Then we reviewed literatures and searched BioGrid 4.4 database (Additional file 2: Fig. S7C), found that Piezo1 might interact with Rab5c, an isoform of Rab5, which belongs to the small GTPases of Rab family. Double IF revealed the colocalization of Piezo1 and Rab5c in HCCLM3^NC^ and Hep3B^NC^ cells. More than that, we also observed that the Rab5c has a tendency to enriched in membrane when Piezo1 highly expressed (Fig. 6A). Then, co-IP results revealed Piezo1 and Rab5c could interact with each other in HCC cells (Fig. 6B, C). Next, we investigated whether the interaction between Piezo1 and Rab5c could affect rab5c expression in HCC cells. Interestingly, we found that silenced Piezo1 in HCCLM3 and Hep3B cells did not changed the protein expression level of Rab5c, but the phosphorylation level of Smad2/3 and expression level of EMT markers have changed correspondingly (Fig. 6D). Combined with the location of Rab5c in the IF result, we further analyzed membrane enrichment of Rab5c by western blotting, and confirmed that Rab5c was significantly enriched in membrane fractions in cells highly expressed Piezo1 (Fig. 6E). Summarily, these results indicate that Piezo1 activates TGF-β/Smad2/3 signaling by recruiting Rab5c without change the total expression level of Rab5c.

### Piezo1 mediated activating of TGF-β signaling and promote HCC progression and EMT through Rab5c

**Fig. 7:**
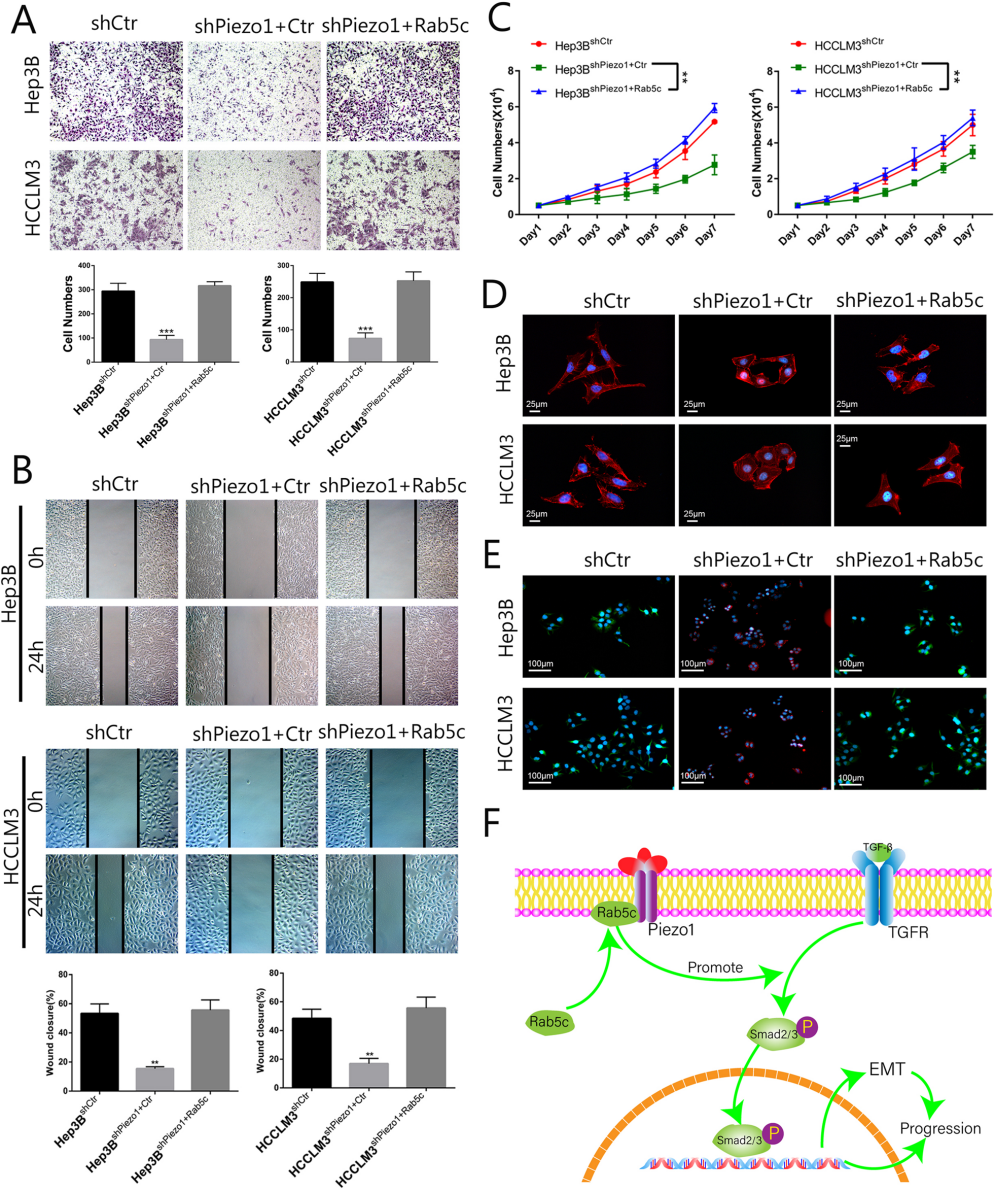
Piezo1 mediated activating of TGF-β signaling and promotion of HCC proliferation, metastasis and EMT through Rab5c. **A** Transwell invasion of HCC cells with interfered expression of piezo1 and ectopic expression of Rab5c (n = 6, **, *P* < 0.01), and analyzed using Student’s t test. **B** Wound healing assays of HCC cells with interfered Piezo1 and Rab5c (n = 6, **, *P* < 0.01), and analyzed using Student’s t test. **C** MTT assays were performed to test the influence of Rab5c on the proliferation of HCC cells with interfered expression of piezo1 and ectopic expression of Rab5c(n = 6, **, *P* < 0.01.), one-way Anova analysis were used, and examined difference between groups with LSD test. **D** Cytoskeleton of HCC cells with interfered expression of piezo1 and ectopic expression of Rab5c. **E** EMT marker expression in piezo1 and Rab5c interfered HCC cells by IF. **F** Schematic depiction of Piezo1 promotes progression of HCC activating through TGF-β signaling by recruiting Rab5c

It has proved that Piezo1 activates TGF-β signaling by recruiting Rab5c in the level of protein, but the function of Rab5c in this progress still unknown. Next, we need to test whether Rab5c is indispensable for Piezo1-mediated activation of TGF-β signaling and promotion of HCC progression and EMT. we transfected Rab5c in HCCLM3^shPiezo1^ and Hep3B^shPiezo1^ cells. MTT, wound healing and transwell assay results revealed overexpression of Rab5c reversed the down-regulation of the invasion, migration and proliferation potential caused by Piezo1 silencing in HCCLM3^shPiezo1^ and Hep3B^shPiezo1^ cells (Fig. 7A–C). The morphology and expression of EMT marker also reversed along with the overexpression of Rab5c in Piezo1 interfered HCC cells (Fig. 7D, E). In summary, the results indicate that Piezo1 promote HCC progression by activating TGF-β signaling via recruiting Rab5c (Fig. 7F).

## Discussion

Recently, considerable achievements have made in the diagnosis and treatment of HCC, such as the advances in surgical treatment, precise treatment and immunological therapy, prolonged survival time of HCC patients to some extent, but invasion and metastasis are still main reason for cancer associated death in HCC. The clinical outcome in HCC patients remains far from satisfactory because of the invasion and metastasis. Thus, it is still critical to gain a better understanding of the mechanisms underlying HCC progression.

Piezo proteins are considered to be large integral membrane proteins with 24–40 transmembrane domains, making them the proteins with the largest number of transmembrane domains [16, 36, 37], and proved as central in diverse biological processes, including cardiovascular development and cancer progression [21, 27, 38]. Piezo1 has been previously verified to be a regulator of various key biological processes, including cell division, migration, and differentiation [39]. But in cancer, the role of Piezo1 is controversial. It has been proved that Piezo1 was downregulated in small lung cancer cell lines, therefore Piezo1 might be a cancer suppressor which suggested to inhibit the cells migration and distant metastases in lung cancer in lung cancer [40]. On the contrary, more reports reminded that Piezo1 might function as an oncogene-related molecule in several types of cancer [26, 28, 41–43]. Piezo1 is highly expressed in the cytoplasm of human prostate carcinoma tissue [44]. In oral squamous cell carcinoma, elevated Piezo1 induced by YAP signaling was required for cell proliferation [42]. Piezo1 is also a vital regulator of innate immune responses and targeting Piezo1 in myeloid cells is protective against cancer with a reduced infiltrate of immune cells [39].

Consistent with majority researches and TCGA, GEO database, we confirmed Piezo1 high expression indicated the poor prognosis in HCC patients after liver resection. Notably, the expression level of Piezo1 was proved to also an independent risk factor for overall survival and disease-free survival of HCC patients. Then, we proved that Piezo1 was associated with poor prognosis of HCC patients. These findings implicate that Piezo1 has potential to serve as an independent prognostic marker for HCC patients after liver resection, which might facilitate precision medicine, helping to predict the prognosis, direct the individualized therapy or act as a therapeutic target.

In order to explore the expression differences for different clinical subtypes of HCC, we have tested the Piezo1 expression level in the 3 subtypes of HCC. It's worth noting that Piezo1 are differently expressed in SLHCC, SHCC and NHCC, the results were also consistent with our previous studies that different clinical HCC subtypes had distinct molecular characteristics [6, 34, 45–49], and Piezo1 might be an marker of molecular subtyping after a large size validation. The functional experiments also revealed that Piezo1 knockdown could inhibit progression of HCC, which identified the function of Piezo1 in promoting HCC aggression. Although Piezo1 proved that might activate various pathways, such as Akt/mTOR pathway [41],MT1-MMP/MMP2 signaling pathway[50], HIF‑1α‑VEGF signaling pathway[43]. Our research showed that Piezo1 activated TGF-β signaling which has not been reported, indicated Piezo1 might be a regulator of multi signaling pathways, and functioning through different pathways in various types of cancers.

In our research in vitro, we have noticed the morphologic change of the shPiezo1 cells, and the GSEA report also reminded us the correlation of Piezo1 and EMT, then we guess that Piezo1 might play a role in EMT of HCC, and the follow-up experiment results established our hypothesis. EMT is one of the key mechanisms of TGF-β signaling regulating cancer progression [51–53]. Lately, one research has proved that downregulated Piezo1 could impairs HCC growth via deregulation of the MAPK-mediated YAP signaling pathway in HepG2 cell line and in *Vivo* [54]. Unlikely, our research revealed the prognostic value of Piezo1 and more concern about invasion and migration of HCC, and GSEA in our research indicates that EMT and TGF-β signaling regulated by Piezo1, but not MAPK or YAP signaling, and we also verified it in clinical specimens. The difference indicates the complex function of Piezo1.

As reported, tumor cells that have lost the cytostatic response may undergo epithelial-to mesenchymal transition (EMT) in response to TGF-β signaling and become more invasive[55]. As one of the hallmarks of signaling pathways that regulate cancer progression, TGF-β signaling induces tumor proliferation and metastasis [56, 57], which also validated in HCC cells of our research. Members of the TGF-β family control numerous cellular functions including proliferation, migration, apoptosis, differentiation, and EMT[58]. Consistent with reports, we found that TGF-β signaling and EMT were both enriched in Piezo1 high group in GSEA, and the result of 10-Pathway Reporter Array further confirmed the GSEA results. Subsequently, we found that Piezo1 knockdown had the equal blockage effect as the specific inhibitor of TGF-β signaling, LY2109761. Our data demonstrated that Piezo1 promote proliferation, invasion and migration of HCC cells through Smad2/3, the canonical TGF-β signaling pathway.

Subsequently, we explored how did Piezo1 regulated TGF-β signaling pathway. In present study, as the BioGrid database indicated, Piezo1 was identified to direct bonding to Rab5c, which is a small GTPase belongs to the Ras-superfamily. It has been shown that Piezo1 could recruits the small GTPase R-Ras to the endoplasmic reticulum (ER), had only a partial effect on H-Ras localization and no effect on other GTPase, in Chinese hamster ovary (CHO) cells, this process might relate to the C-terminus of R-RAS [40]. Normally, TβRII was internalized and a fraction of it was sorted from early endosomes to lysosomes for degradation [59]. Rab5c is a critical regulator of endosomes to lysosomes [60], which indicates that Pieoz1 might affect TGF-β signaling through decreased degradation of TβRII when Rab5c was recruited to membrane. Rab5c was determined to be involved in cell proliferation, transformation, survival and metastasis in types of cancers through various signaling pathways [33–35], and it was also upregulated in transcriptional level and was recruited more to cell membrane of Marfan vascular smooth muscle cells (VSMC) and activated TGF-β signaling [29]. In this study, we found that Rab5c has a trend to located in membrane while Piezo1 highly expressed, then we confirmed that Rab5c was significantly enriched in membrane fractions of HCC cell lines. Therefore, our results indicated that Rab5c was the potential target of Piezo1 in HCC. In HCCLM3^shPiezo1^ and Hep3B^shPiezo1^ cells, silencing of Piezo1 decreased activation of TGF-β signaling. Meanwhile, Rab5c ectopic expression could regain the activity of TGF-β signaling, and recovered the invasiveness, migrations, and mesenchymal phenotype in Piezo1 knockdown HCC cells. Although previous studies have confirmed that Rab5c was an oncogene that promote cancer progression or chemoresistance through various pathways [61–63], but our research first illustrate that Rab5c act as a target of Piezo1 and activate TGF-β signaling through in cancer cells.

Even we prefer to perfect our research design, but there are several unavoidable limitations. Our research explored the potential prognostic value of Piezo1in HCC through two independent cohort and TCGA database, in protein and mRNA level, but the long term, multicenter and large sample size studies are important factors to identify a functional biomarker. In mechanism, we have identified that Ca^2+^ influx was not the dominant factor in Piezo1 activated TGF-β signaling in HCC cells, but the significance of Ca^2+^ influx in vivo or cellular Ca^2+^ homeostasis in HCC patients still worth investigating. The function and mechanism of Piezo1 in HCC were exerted, the regulator of Piezo1 expression level in HCC and how to intervene Piezo1 in HCC to obtain better prognosis remains our further research.

## Conclusion

In conclusion, our study demonstrated that Piezo1 is highly expressed and is significantly correlated with poor prognosis in HCC. Piezo1 has potential to serve as a risk predicting marker for independent prognostic indicator for HCC patients. Moreover, we have identified that Piezo1 promoted HCC progression and EMT through activating TGF-β signaling by recruiting Rab5c. Our finding also indicate that Piezo1 can be used as a novel prognostic biomarker and a potential therapeutic target in HCC.

## Supplementary Information

**Additional file 1.** Methods and materials.

**Additional file 2: Fig. S1.** Piezo1 and Piezo2 protein quantification in western blotting shown in Fig1. A (n=3,*P<0.05, **P<0.01,***P<0.001). **Fig. S2.** Interfered Piezo1 in HCCLM3 and Hep3B cells. (A-B) After interfered Piezo1 in HCCLM3 and Hep3B cells by thre e shRNAs separately, the expression level of Piezo1 was identified by real-time PCR(A) and western blotting(B). **Fig. S3.**MTT assays was used to detect the proliferation of Hep3BshPiezo1, HCCLM3shPiezo1 and the control HCC cells (n=6 for each group). **Fig. S4.** Growth curve of subcutaneous xenograft tumor derived Hep3BshPiezo1, HCCLM3shPiezo1 and the control HCC cells (n=6 each group, **P<0.01). **Fig. S5.** Weight of subcutaneous xenograft tumors derived Hep3BshPiezo1, HCCLM3shPiezo1 and the control HCC cells(n=6 each group, **P<0.01). **Fig. S6.** Ki67 for the subcutaneous xenograft tumors and orthotopic xenograft tumors was detected by IHC. **Fig. S7.** Ca2+ influx was not the dominant factor in Piezo1 activated TGF-β signaling. (A) Measurements of Ca2+ concentration after treated HCCLM3 and Hep3B cells with Yoda1(the activator of Piezo1, 20mM) and GsMTx4 (the inhibitor of Piezo1, 2.5μM). (B) Examination of p-Smad2/3 and marker of EMT by Western blot, the Ca2+ influx was not the dominant factor in Piezo1 activated TGF-β signaling and EMT. (C) BioGrid 4.4 database indicates that Piezo1 might interact with Rab5c.

**Additional file 3: Table S1.** Clinicopathologcal characteristics of HCC patients in training cohort and validation cohort. **Table S2.** Univariate and multivariate analyses of risk factors associated with overall survival and disease-free survival of HCC patients in validation cohort. **Table S3.** High expressed pathways in the result of Gene set enrichment analysis (GSEA). **Table S4.** Low expressed pathways in the result of Gene set enrichment analysis (GSEA). **Table S5.** Volume data of the orthotopic xenograft tumors in Fig. 3F.

## Data Availability

All data that support this study are available from the corresponding author upon reasonable request.
