# Supplementary material for: Piezo1 promoted hepatocellular carcinoma progression and EMT through activating TGF-β signaling by recruiting Rab5c
Source: Cancer Cell Int. 2022 Apr 23;22:162. doi: 10.1186/s12935-022-02574-2 (PMC9035260; doi:10.1186/s12935-022-02574-2)
Supplement: Supplementary file 1 — Additional file 1. Methods and materials. [file 12935_2022_2574_MOESM1_ESM.pdf]

Supplementary methods and materials:

Cell lines and Cell Culture

MHCC97-L, MHCC97-H and HCCLM3 were kindly provided by the Liver Cancer Institute of Fudan University, Shanghai, China. PLC/PRF/5, Hep3B and HepG2 cells were purchased from the American Type Culture Collection (ATCC, Manassas, VA). SMMC7721 and Huh7 cells were purchased from the Cell Bank of Typical Culture Preservation Committee of Chinese Academy of Science, Shanghai, China. Cells were maintained in Dulbecco's Modified Eagle's Medium (Biological Industries, Kibbutz Beit Haemek, Israel) supplemented with 10% fetal bovine serum, 100 μg/mL streptomycin, and 100 U/mL penicillin (Hyclone, Logan, UT) at 37 °C in a humidified atmosphere with 5% CO2.

Isolation of Primary Human Hepatocytes (PHH).

All procedures were performed in accordance with the provision of the Ethical Commission of Xiangya Hospital of Central South University. Primary human hepatocytes were isolated from normal liver tissues, which were obtained from tissues far away from liver hemangioma tissues. Primary human hepatocytes were isolated and cultured as described [1,2].

Protein extraction and western blot

Tissues or cells were lysed with RIPA buffer (Pierce, Rockford, IL) supplemented with protease inhibitors. Protein concentration was measured using a BCA protein assay (Thermo Scientific, Rockford, IL). Protein lysates, suspended in loading buffer, were separated on 10% SDS-polyacrylamide gels and transferred onto PVDF membranes (Millipore, Belford, MA). Then these membranes were blocked with 5% skim milk at room temperature for 1 h, and incubated with primary antibodies at 4 °C overnight. After washed, they were incubated with suitable HRP-conjugated secondary antibody at room temperature for 30 min and detected using an enhanced chemiluminescence (ECL) kit (Thermo Scientific). Antibodies for Piezo1, Smad2/3 and p-Smad2/3 were obtained from Abcam (Cambridge, MA), for Smad2/3 and p-Smad2/3, E-cadherin, and Vimentin were purchased from Santa Cruz Biotechnology (Santa Cruz, CA), β-actin and GAPDH antibody and corresponding secondary antibodies were purchased from Zhongshan Golden Bridge Biotechnology (ZSGB, Beijing, China).

RNA extraction and quantitative real-time PCR

Total RNA was extracted from tissues or cells with TRIzol reagent (Invitrogen, Carlsbad, CA). Reverse transcription were performed using an Advantage RT-for-PCR Kit (Takara, Dalian, China). qRT-PCR analysis was done using SYBR®Green Real time PCR Master Mix assay kit (Takara) in a 7300 Real-Time PCR system (Applied Biosystems Inc., Foster City, CA) with the following primers(5'→3'): Piezo1 forward, TGCAGCCGAGAGACAGAGAAGAAA and reverse, ACA TGAAGAGCAGTGGGAACCAGA; Rab5c forward, CCGAACAAGCAAAGAACAGTG and reverse, GACGCAGCATCAGTATTCCAAT. GAPDH was used as endogenous control.

Transfection and clone selection

The ectopic expression and knockdown lentivirus as well as control lentivirus for Piezo1 and Rab5c were all purchased from GenePharma (Suzhou, China). Transfection was performed according to the manufacturer's instructions. Puromycin (2 μg/mL) was used to select stable clones. The

sequences of shRNA are listed as follows (5′→3′): Piezo1-sh1: GCCGTCACTGAAGATGTAAAC; Piezo1-sh2: GCCAGTTCTACGGACAGAAGA; Piezo1-sh3: GCCCAGCAACGAATCAAATGG; Rab5c-sh1: ATCAATTCAAGAGATTGATGT; Rab5c1-sh2: ACT TTTTCAAGAGAAAAGTTC; Rab5c-sh3: CAATATTCA AGAGATATTGTT.

MTT assay and colony formation assay

For MTT assay, 5 × 103 cells were seeded in 96-well plates, incubated for 0–7 days, stained with MTT, and absorbance values were determined at 570 nm. The relative cell number was normalized by the absorbance from the control cells. For colony formation assays, 500 cells were seeded per well in 6-well plates and cultured for 14 days. The colonies were fixed with 4% paraformaldehyde and stained with 1% crystal violet. Only colonies containing more than 50 cells were counted.

Wound healing and transwell invasion assay

For wound healing assay, 5 × 105 cells were seeded into 6-well plates and grown to confluence. Mitomycin C (10 μg/mL) was used to suppress cell proliferation before scratching [27]. Wounds were created by scraping the confluent cell monolayers with a 10 μL pipette tip. After extensively rinsed to remove cellular debris, cells were cultured in serum-free medium. The wound closure rate was monitored every 12 h and images were taken using an inverted microscope TE-2000S (Nikon, Tokyo, Japan). Transwell invasion assay was performed in a 24-well transwell plate with 8-μm polyethylene terephthalate membrane filters (Corning Costar Corp, Corning, NY). 1×105 cells in 200 μL of serum-free medium were added to the upper chambers, which containede Matrigel coated membranes (BD Biosciences). Each lower chamber was filed with 500 μL medium with 10% FBS. After 18 h or 24 h of incubation, cells that invaded to the bottom chamber were fixed with 4% paraformaldehyde and stained with 0.1% crystal violet. Invasive cells were counted in five randomly chosen fields (magnification, × 200) per well.

Cancer 10-pathway reporter arrays

A Cignal Finder 10-Pathway Reporter Array (SABiosciences, Valencia, CA) was performed to explore the signaling pathways that were regulated by Piezo1 in HCC cells. The assay was conducted according to the manufacturer's protocol. Relative firefly luciferase activity was calculated and normalized to the constitutively expressed Renilla luciferase. Experiments were done in triplicates.

Gene set enrichment analysis (GSEA)

The GSEA (http://www.broadinstitute.org/gsea) tool was used to determine the differential enrichment of gene sets in the HCC patient samples belonging to high and low Piezo1 expression groups. Gene expression data from TCGA were divided into high and low expression groups according to the median level of Piezo1 mRNA expression, and GSEA was used to explore the influence of Piezo1 expression level on each gene and to analyze the mechanism underlying the involvement of Piezo1 in the invasion and metastasis of HCC. The genome was sequenced 1000 times per analysis. In addition, the level of Piezo1 was used as a phenotypic marker. The nominal p-value (NOM p) and the normalized enrichment score (NES) were used to classify enrichment pathways in each phenotype.

Intracellular Ca^{2+} measurements

The measurements were performed using Fura-2 AM (Abcam, Cambridge, UK) as described in protocol. After treated with Yoda1(20 mM, R&D) or GsMTx4(2.5μM, Abcam), 5 × 10^4 cells were suspended in HBSS buffer containing 4 μM Fura-2 AM and loaded onto a 96-well black plate for 1 h at 37 °C. Following an internal Ca^{2+} depletion by 2 μM the apsigargin for 10 min, 2 μM extracellular Ca2+ was added and Fura-2 AM signals were measured using a Multimode Microplate Readers (VICTOR Nivo, PerkinElmer, Waltham, MA, USA) at 340/510 and 380/510 nm.

Co-immunoprecipitation (co-IP) assay

For Co-IP, pre-cleared protein from whole cell lysates were incubated with antibody against Piezo1 or Rab5c at 4 °C overnight, which was conjugated to AminoLink Plus Resin (Pierce, Rockford, IL), The IP targets were disassociated from the immobilized antibodies on the AminoLink Plus Resin by the gentle elution buffer. Eluted proteins were resolved using 10% SDS-PAGE, followed by western blot with appropriate antibodies.

Cell immunofluorescence staining

Indicated HCC cells (2 × 10^4 cells) were seeded into 12-well plate with glass coverslips for 24 h. Then cells were successively fixed in 4% paraformaldehyde, permeabilized with 0.2% Triton X-100, blocked with 1% BSA and incubated with primary antibody at 4 °C overnight. After washed with PBS, cells were incubated with appropriate DyLight-conjugated secondary antibody and DAPI (Vector laboratories, Burlingame, CA). Finally, the slides were mounted and images were captured using an inverted fluorescence microscope DMI4000-B (Leica, Wetzlar, Germany).

Table. 1 The sequences of PCR primers used in this study

Genes	Application	Sequence (5'→3')
Piezo1	qRT-PCR	F: TGC AGC CGA GAG ACA GAG AAG AAA
		R: ACA TGA AGA GCA GTG GGA ACC AGA
GAPDH	qRT-PCR	F: TGGCCTTCCGTGTTCCTAC
		R: GAGTTGCTGTTGAAGTCGCA
Piezo2	qRT-PCR	F:ATTGGCTGGAGGAGAAA
		R:GAAGGTGGAAGAGTGGGAGT

Table.2. The primary antibodies used in this study

Primary Antibodies	Origin	Application	Dilution
Piezo1	Proteintech (10294-2-AP)	IHC	1:50
		WB	1:500
		IP	1:300
		IF	1:200

Piezo2	Proteintech (26205-1-AP)	IHC	1:50
		WB	1:500
E-cadherin	CST (#14472)	WB	1:1000
		IF	1:200
		IHC	1:100
Vimentin	CST (#5741)	WB	1:1000
		IF	1:100
		IHC	1:200
Snail	CST(#3895)	WB	1:500
Erk1/2	CST(#9107)	WB	1:500
p-Erk1/2	CST(#4370)	WB	1:500
Akt	CST (#14702)	WB	1:500
p-Akt	CST (#4058)	WB	1:500
p-Smad2	Affinity (AF3449)	IHC	1:400
Smad2/3	CST (#3102)	WB	1:1000
p-Smad2/3	CST (#8828)	WB	1:1000
Rab5c	SantaCruz(sc-365667)	WB	1:1000
TrfR	Affinity (DF6250)	WB	1:1000
RhoGDI	Affinity (DF7414)	WB	1:1000
IgG1	CST (#5415)	Isotype Control	1:300
β-actin	ProteinTech (60008-1-Ig)	WB	1:5000
GAPDH	Affinity (AF0863)	WB	1:1000

Table.3 List of secondary antibodies and reagents used in this study

**Secondary antibodies or reagents**	**Origin**	**Application**	**Dilution**
Goat anti-Rabbit IgG (H+L) Secondary Antibody, DyLight 594 conjugate	ThermoFisher Scientific (#35560)	IF	1:100
Goat anti-Rabbit IgG (H+L) Secondary Antibody, DyLight 488	ThermoFisher Scientific (#35553)	IF	1:100

conjugate			
Goat anti-Mouse IgG (H+L) Secondary Antibody, DyLight 594 conjugate	ThermoFisher Scientific (#35510)	IF	1:100
Goat anti-Mouse IgG (H+L) Secondary Antibody, DyLight 488 conjugate	ThermoFisher Scientific (#35503)	IF	1:100
Mitomycin C	Roche (M8170)	/	10 μg/ml
LY2109761	Selleck(S2704)	/	5mg/ml
DMSO	Solarbio(D8371)	Solvent	/

Table. 4. The sequences of RNAi used in this study

Name	Sequence
Piezo1-shRNA1	5'- GACUACUUCCUGUUUGAGUCC -3'
Piezo1-shRNA2	5'- GUCUCAAGAACUUCGUAGATT -3'
Piezo1-shRNA3	5'- GCCUCGUGGUCUACAAGAUTT -3'
Rab5c-shRNA1	5′-GGACAGGAGCGGUAUCACA-3′
Rab5c-shRNA2	5′-AATGAACGTGAACGAAATCTT-3′
Rab5c-shRNA3	5′-ACCAACACAGATACATTTGCA-3′

Reference:

1. Fang F, Chang RM, Yu L, et al. MicroRNA-188-5p suppresses tumor cell proliferation and metastasis by directly targeting FGF5 in hepatocellular carcinoma. *J Hepatol* 2015;63(4):874-85. doi: 10.1016/j.jhep.2015.05.008 [published Online First: 2015/05/23]
2. Zhu H, Elyar J, Foss R, et al. Primary human hepatocyte culture for HCV study. *Methods Mol Biol* 2009;510:373-82. doi: 10.1007/978-1-59745-394-3_28
