# Supplementary material for: Piezo1 promoted hepatocellular carcinoma progression and EMT through activating TGF-β signaling by recruiting Rab5c
Source: Cancer Cell Int. 2022 Apr 23;22:162. doi: 10.1186/s12935-022-02574-2 (PMC9035260; doi:10.1186/s12935-022-02574-2)
Supplement: Supplementary file 2 — Additional file 2: Fig. S1. Piezo1 and Piezo2 protein quantification in western blotting shown in Fig1. A (n=3,*P<0.05, **P<0.01,***P<0.001). Fig. S2. Interfered Piezo1 in HCCLM3 and Hep3B cells. (A-B) After interfered Piezo1 in HCCLM3 and Hep3B cells by thre e shRNAs separately, the expression level of Piezo1 was identified by real-time PCR(A) and western blotting(B). Fig. S3.MTT assays was used to detect the proliferation of Hep3BshPiezo1, HCCLM3shPiezo1 and the control HCC cells (n=6 for each group). Fig. S4. Growth curve of subcutaneous xenograft tumor derived Hep3BshPiezo1, HCCLM3shPiezo1 and the control HCC cells (n=6 each group, **P<0.01). Fig. S5. Weight of subcutaneous xenograft tumors derived Hep3BshPiezo1, HCCLM3shPiezo1 and the control HCC cells(n=6 each group, **P<0.01). Fig. S6. Ki67 for the subcutaneous xenograft tumors and orthotopic xenograft tumors was detected by IHC. Fig. S7. Ca2+ influx was not the dominant factor in Piezo1 activated TGF-β signaling. (A) Measurements of Ca2+ concentration after treated HCCLM3 and Hep3B cells with Yoda1(the activator of Piezo1, 20mM) and GsMTx4 (the inhibitor of Piezo1, 2.5μM). (B) Examination of p-Smad2/3 and marker of EMT by Western blot, the Ca2+ influx was not the dominant factor in Piezo1 activated TGF-β signaling and EMT. (C) BioGrid 4.4 database indicates that Piezo1 might interact with Rab5c. [file 12935_2022_2574_MOESM2_ESM.pdf]

Supplementary Figures

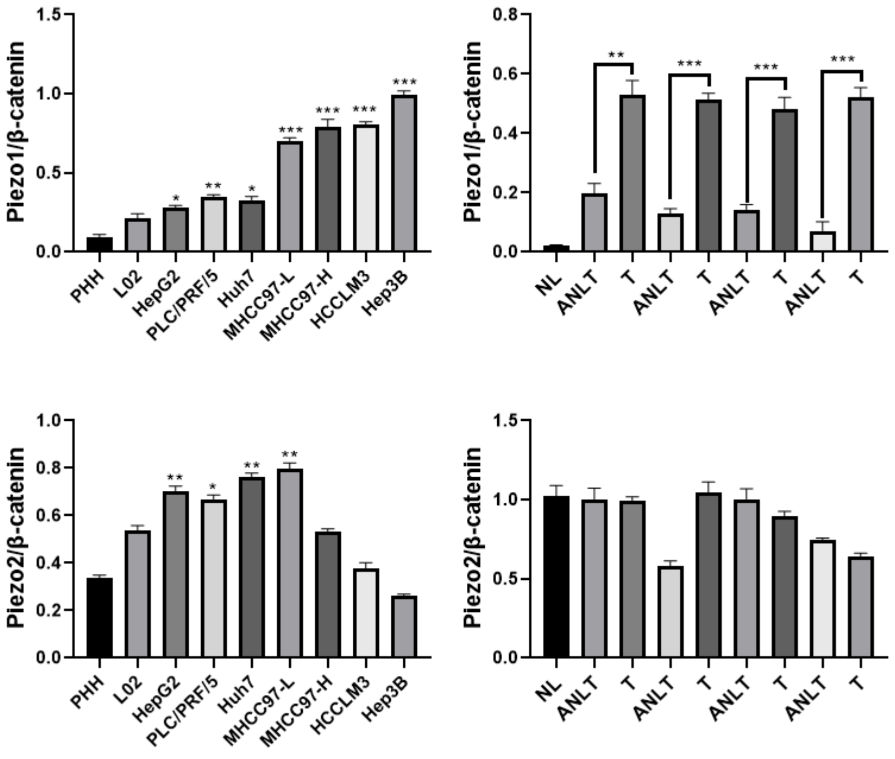

Supplementary Fig. S1 Piezo1 and Piezo2 protein quantification in western blotting shown in Fig1. A (n=3,*P<0.05, **P<0.01,***P<0.001).

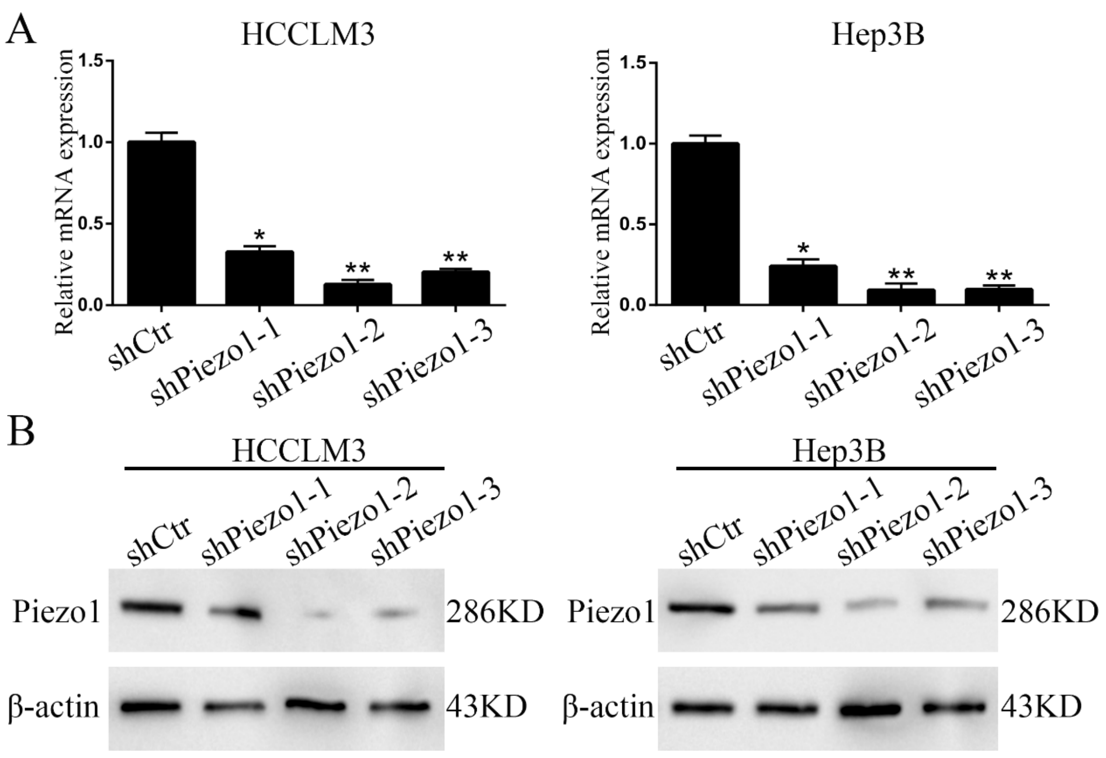

Supplementary Fig. S2 Interfered Piezo1 in HCCLM3 and Hep3B cells. (A-B) After interfered Piezo1 in HCCLM3 and Hep3B cells by three shRNAs separately, the expression level of Piezo1 was identified by real-time PCR(A) and western blotting(B).

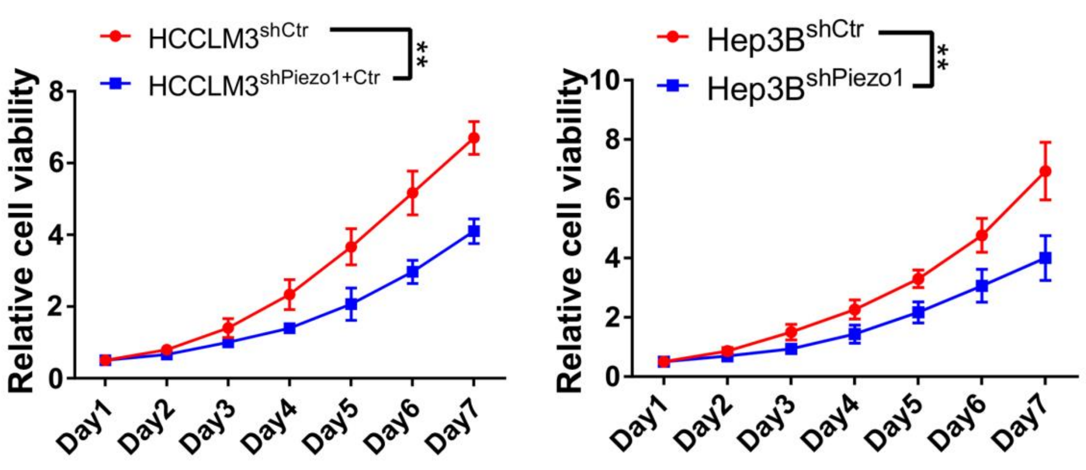

Supplementary Fig. S3 MTT assays was used to detect the proliferation of Hep3B^{shPiezo1}, HCCLM3^{shPiezo1} and the control HCC cells (n=6 for each

group).

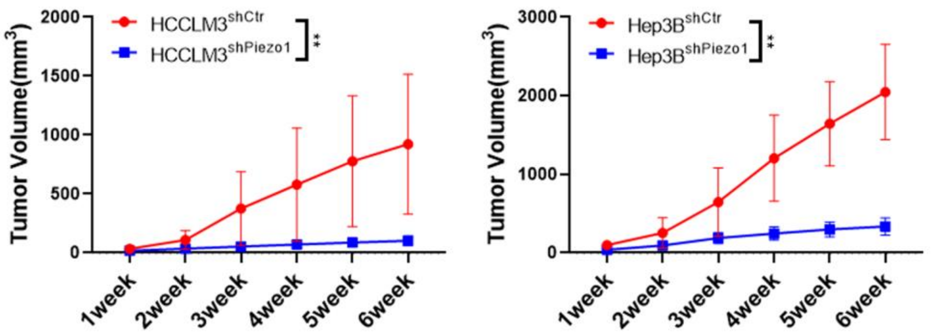

Supplementary Fig. S4 Growth curve of subcutaneous xenograft tumor derived Hep3B^{shPiezo1}, HCCLM3^{shPiezo1} and the control HCC cells (n=6 each group, **P<0.01).

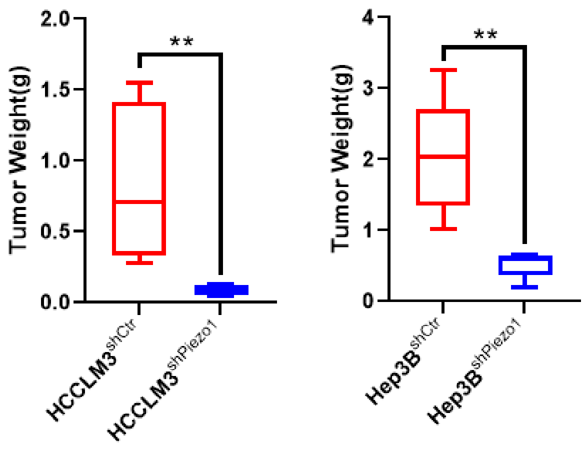

Supplementary Fig. S5 Weight of subcutaneous xenograft tumors derived Hep3B^{shPiezo1}, HCCLM3^{shPiezo1} and the control HCC cells(n=6 each group, **P<0.01)

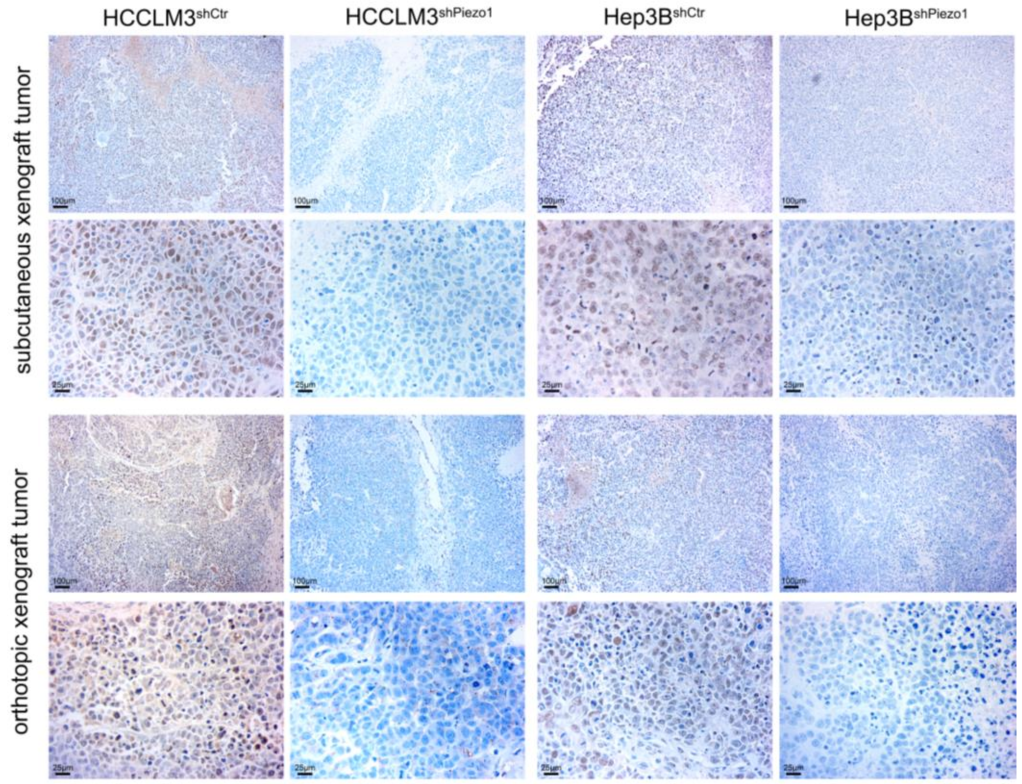

Supplementary Fig. S6 Ki67 for the subcutaneous xenograft tumors and orthotopic xenograft tumors was detected by IHC.

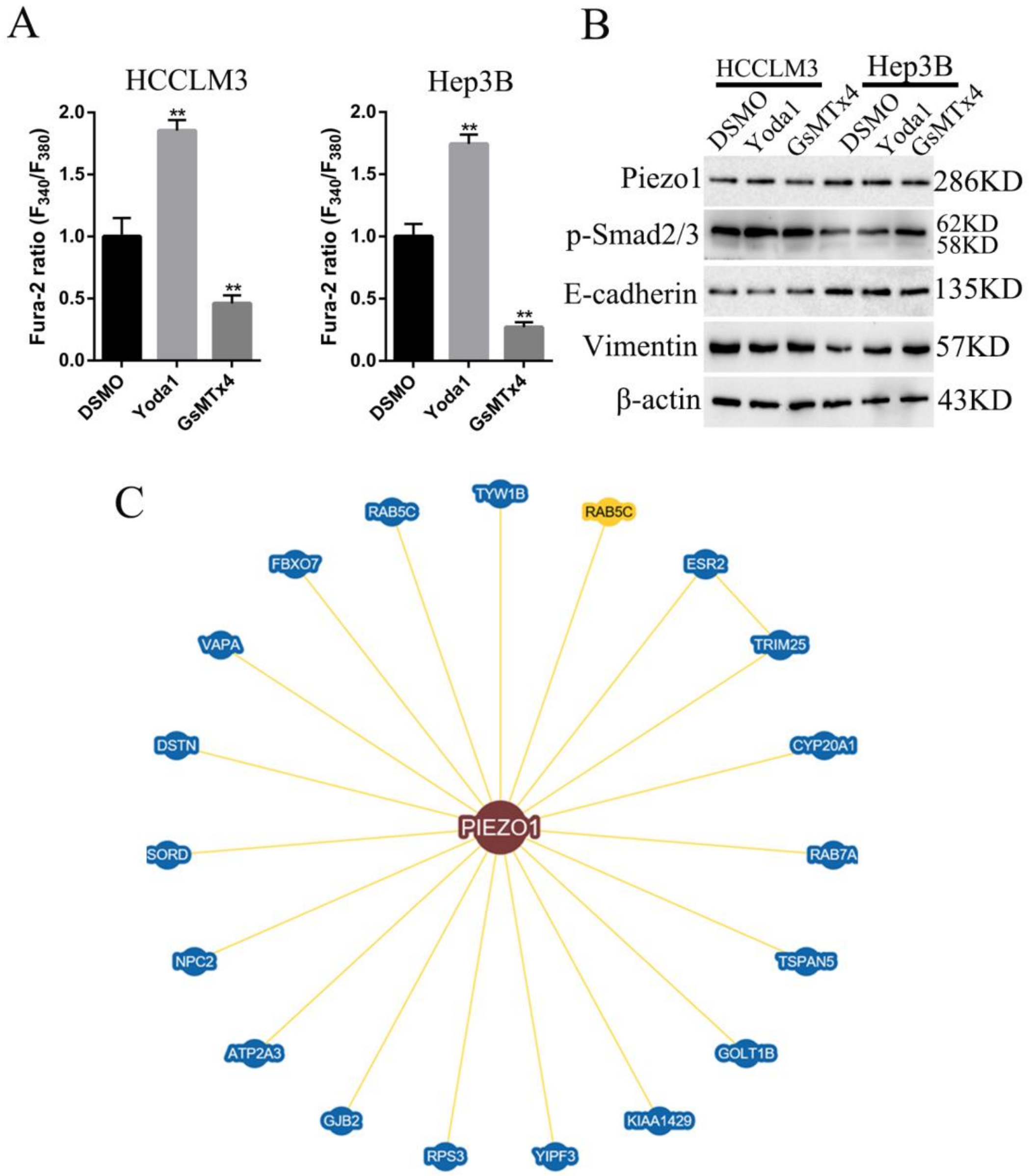

Supplementary Fig. S7 Ca^{2+} influx was not the dominant factor in Piezo1 activated TGF-β signaling. (A) Measurements of Ca^{2+} concentration after treated HCCLM3 and Hep3B cells with Yoda1(the activator of Piezo1, 20mM) and GsMTx4 (the inhibitor of Piezo1, 2.5μM). (B) Examination of p-Smad2/3 and marker of EMT by Western blot, the Ca^{2+} influx was not the dominant factor in Piezo1 activated TGF-β signaling and EMT. (C) BioGrid 4.4 database indicates that Piezo1 might interact with Rab5c.
