# Supplementary material for: Piezo1 promoted hepatocellular carcinoma progression and EMT through activating TGF-β signaling by recruiting Rab5c
Source: Cancer Cell Int. 2022 Apr 23;22:162. doi: 10.1186/s12935-022-02574-2 (PMC9035260; doi:10.1186/s12935-022-02574-2)
Supplement: Supplementary file 3 — Additional file 3: Table S1. Clinicopathologcal characteristics of HCC patients in training cohort and validation cohort. Table S2. Univariate and multivariate analyses of risk factors associated with overall survival and disease-free survival of HCC patients in validation cohort. Table S3. High expressed pathways in the result of Gene set enrichment analysis (GSEA). Table S4. Low expressed pathways in the result of Gene set enrichment analysis (GSEA). Table S5. Volume data of the orthotopic xenograft tumors in Fig. 3F. [file 12935_2022_2574_MOESM3_ESM.pdf]

Supplementary Table S1. Clinicopathologcal characteristics of HCC patients in training cohort and validation cohort.

Clinicopathologic variables	Counts		*P*
	Training cohort	Validation cohort	
**Gender**			0.430
Female	42(28.00%)	31(23.85%)	
Male	108(72.00%)	99(76.15%)	
**Age (years)**			0.525
≤50	67(44.67%)	63(48.46%)	
>50	83(55.33%)	67(51.54%)	
**AFP(**ng/ml**)**			0.322
<20	36(24.00%)	38(29.23%)	
≥20	114(76.00%)	92(70.77%)	
**Hepatitis B status**			0.141
Negative	43(28.67%)	48(36.92%)	
Positive	107(71.33%)	82(63.08%)	
**Liver cirrhosis**			0.535
Absent	69(46.00%)	55(42.31%)	
Present	81(54.00%)	75(57.69%)	
**Child-Pugh classification**			0.599
A	98(65.33%)	81(62.31%)	
B	52(34.67%)	49(37.69%)	
**Tumor size (cm)**			0.463
≤ 5	57(38.00%)	55(42.31%)	
> 5	93(62.00%)	75 (57.69%)	
**Tumor nodule number**			0.269
Solitary	72(48.00%)	71(54.62%)	
Multiple (≥ 2)	78(52.00%)	59(45.38%)	
**Capsulation formation**			0.454
Presence	58(38.67%)	56(43.08%)	

Absence	92(61.33%)	74(56.92%)	
**Edmondson-Steiner grade**			0.972
Ⅰ&Ⅱ	62(41.33%)	54(41.54%)	
Ⅲ&Ⅳ	88(58.67%)	76(58.46%)	
**Microvascular invasion**			0.584
Absence	91(60.67%)	83(63.85%)	
Presence	59(39.33%)	47(36.15%)	
**Macrovascular invasion**			0.704
Absence	122(81.33%)	108(83.08%)	
Presence	28(18.67%)	22(16.92%)	
**BCLC stage**			0.285
0&A	43(28.67%)	45(34.62%)	
B&C	107(71.33%)	85(65.38%)	
**TNM stage**			0.768
Ⅰ	62(41.33%)	56(43.08%)	
Ⅱ&Ⅲ	88(58.67%)	74(56.92%)	
**CNLC stage**			0.398
Ⅰ	86(57.33%)	81(62.31%)	
Ⅱ&Ⅲ	64(42.67%)	49(37.69%)	

Abbreviations: AFP, alpha-fetoprotein; HBsAg, hepatitis B surface antigen; TNM, tumor node metastasis; BCLC, Barcelona Clinic Liver Cancer; CNLC, China Clinic Liver Cancer.

Supplementary Table S2. Univariate and multivariate analyses of risk factors associated with overall survival and disease-free survival of HCC patients in validation cohort.

**Clinicopathol o-gic**	**OS**		**DFS**	
	**Univariate Analysis**	**Multivariate Analysis**	**Univariate Analysis**	**Multivariate Analysis**
	P **HR(95% CI)**	***P*** **HR(95% CI)**	***P*** **HR(95% CI)**	***P*** **HR(95% CI)**
**Gender**	0.072	NA	0.051	NA

Female	1		1	
Male	1.574(0.961-2.578)		1.528(0.998-2.339)	
**Age (years)**	0.341	NA	0.628	NA
≤50	1		1	
>50	1.215(0.814-1.815)		1.091(0.766-1.556)	
**AFP(**ng/ml)	**0.001**	NA	0.019	0.009
<20	**1**		1	1
≥20	**2.258(1.387-3.674)**		1.615(1.081-2.412)	1.843(1.165-2.915)
**Hepatitis B status**	0.141	NA	0.185	NA
Negative	1		**1**	
Positive	1.369(0.901-2.078)		**1.276(0.890-1.828)**	
**Liver cirrhosis**	0.121	**NA**	**0.049**	0.525
Absent	1		**1**	1
Present	1.384(0.918-2.086)		**1.440(1.001-2.072)**	1.138(0.764-1.696)
**Child-Pugh classification**	0.331	NA	0.779	NA
A	1		**1**	
B	1.267(0.786-2.040)		**1.063(0.693-1.631)**	
**Tumor size (cm)**	0.202	**NA**	**0.345**	**NA**
≤ 5	1		**1**	
> 5	1.305(0.867-1.966)		**1.187(0.832-1.694)**	
**Tumor nodule number**	**<0.001**	**0.006**	**<0.001**	**<0.001**
Solitary	**1**	**1**	**1**	**1**
Multiple (≥ 2)	**2.141(1.431-3.204)**	**1.963(1.217-3.166)**	**1.934(1.365-2.757)**	**2.375(1.573-3.585)**
**Capsulation formation**	**0.012**	0.524	**0.092**	0.390
Presence	**1**	1	**1**	1
Absence	**1.692(1.122-2.553)**	1.252(0.627-2.497)	**1.356(0.951-1.933)**	1.321(0.700-2.493)
**Edmondson-Steiner grade**	**0.001**	0.083	**0.001**	0.761
Ⅰ&Ⅱ	**1**	1	**1**	1

III&IV	**2.049(1.342-1.327)**	1.523(0.946-2.451)	**1.823(1.272-2.612)**	1.107(0.574-2.134)
**Microvascular invasion**	**<0.001**	**0.019**	**<0.001**	**0.004**
Absence	**1**	**1**	**1**	**1**
Presence	**2.013(1.324-3.059)**	**1.801(1.104-2.940)**	**2.409(1.651-3.515)**	**1.993(1.252-3.171)**
**Macrovascular invasion**	**<0.001**	**0.002**	**<0.001**	**<0.001**
Absence	**1**	**1**	**1**	**1**
Presence	**2.676(1.624-4.408)**	**2.215(1.350-3.635)**	**2.594(1.606-4.191)**	**2.268(1.158-4.442)**
**BCLC stage**	**0.022**	**0.047**	**0.013**	**0.037**
0&A	**1**	**1**	**1**	**1**
B&C	**1.669(1.075-2.591)**	**1.656(1.007-2.722)**	**1.599(1.105-2.315)**	**1.584(1.027-2.443)-**
**TNM stage**	**<0.001**	**0.020**	**0.003**	**0.008**
I	**1**	**1**	**1**	**1**
II&III	**2.263(1.487-3.442)**	**1.784(1.096-2.904)**	**1.754(1.215-2.532)**	**1.818(1.170-2.825)**
**CNLC stage**	**0.022**	**0.017**	**0.032**	**0.046**
I	**1**	**1**	**1**	**1**
II&III	**1.623(1.072-2.459)**	**1.821(1.114-2.977)**	**1.493(1.036-2.151)**	**1.551(1.007-2.388)**
**Piezo1 expression**	**<0.001**	**0.024**	**<0.001**	**0.017**
Low	**1**	**1**	**1**	**1**
High	**2.307(1.487-3.580)**	**1.731(1.074-2.790)**	**1.974(1.363-2.858)**	**1.716(1.100-2.675)**

Supplementary Table S3. High expressed pathways in the result of Gene set enrichment analysis (GSEA).

NAME	NES	NOM p-val
**HALLMARK_TGF_BETA_SIGNALING**	**1.7752404**	**0.001960784**
HALLMARK_APICAL_JUNCTION	1.7562696	0.001968504
HALLMARK_MYOGENESIS	1.7198752	0.001953125
HALLMARK_APICAL_SURFACE	1.6900234	0
**HALLMARK_EPITHELIAL_MESENCHYMAL_TRANSITION**	**1.6779602**	**0.003929273**
HALLMARK_NOTCH_SIGNALING	1.6673367	0.007692308
HALLMARK_WNT_BETA_CATENIN_SIGNALING	1.6370306	0
HALLMARK_UV_RESPONSE_DN	1.6245838	0.007968128
HALLMARK_HEDGEHOG_SIGNALING	1.5995626	0.01171875

HALLMARK_APOPTOSIS	1.5952106	0.015810277
HALLMARK_ESTROGEN_RESPONSE_EARLY	1.5892456	0.011881189
HALLMARK_ANGIOGENESIS	1.58451	0.017274473
HALLMARK_TNFA_SIGNALING_VIA_NFKB	1.5837303	0.026
HALLMARK_INFLAMMATORY_RESPONSE	1.5658875	0.03777336
HALLMARK_IL2_STAT5_SIGNALING	1.5491016	0.01622718
HALLMARK_HYPOXIA	1.5176268	0.041666668
HALLMARK_P53_PATHWAY	1.4977729	0.04024145
HALLMARK_KRAS_SIGNALING_UP	1.4870514	0.034951456

Supplementary Table S4. Low expressed pathways in the result of Gene set enrichment analysis (GSEA).

NAME	NES	NOM p-val
HALLMARK_MYC_TARGETS_V1	-1.6872948	0.09202454
HALLMARK_OXIDATIVE_PHOSPHORYLATION	-1.576084	0.14915967
HALLMARK_DNA_REPAIR	-1.4901795	0.091976516
HALLMARK_BILE_ACID_METABOLISM	-1.3129461	0.19881889
HALLMARK_E2F_TARGETS	-1.2281697	0.33333334
HALLMARK_FATTY_ACID_METABOLISM	-1.2043116	0.29674795
HALLMARK_PEROXISOME	-1.1629767	0.3253493
HALLMARK_XENOBIOTIC_METABOLISM	-1.1613581	0.33197555
HALLMARK_PANCREAS_BETA_CELLS	-1.1024553	0.334004
HALLMARK_COAGULATION	-0.839716	0.6639511
HALLMARK_G2M_CHECKPOINT	-0.781734	0.6812749
HALLMARK_CHOLESTEROL_HOMEOSTASIS	-0.76405555	0.7550201
HALLMARK_SPERMATOGENESIS	-0.6681743	0.96190476
HALLMARK_MYC_TARGETS_V2	-0.6474292	0.78373015

Supplementary Table S5. Volume data of the orthotopic xenograft tumors in Fig3. F

HCCLM3^{shCtr}			HCCLM3^{shPiezo1}			Hep3B^{shCtr}			Hep3B^{shPiezo1}		
L	W	Volum	L	W	Volum	L	W	Volum	L	W	Volum
14.2	10	710	10.6	7.7	314.237	12.9	10.6	724.722	6.5	3.5	39.8125
13.5	7.1	340.2675	5.5	3.2	28.16	14.5	10.6	814.61	6.1	4.8	70.272
13.5	10	675	4.2	3.2	21.504	7.7	5.8	129.514	2.9	2.3	7.6705
9.7	8.1	318.2085	3.2	2.6	10.816	9.7	8.4	342.216	4.8	3.9	36.504
8.7	7.7	257.9115	2.9	2.3	7.6705	11.6	8.1	380.538	2.6	2.3	6.877
12.3	6.5	259.8375	2.3	1.6	2.944	13.9	11.6	935.192	7.1	4.8	81.792

*measured in millimeters (mm)。
